# Filamentous‐Actin‐Mimicking Nanoplatform for Enhanced Cytosolic Protein Delivery

**DOI:** 10.1002/advs.202305600

**Published:** 2023-12-28

**Authors:** Yuqiong Xia, Keyun Wu, Chang Liu, Xuejuan Zhao, Jun Wang, Jianxia Cao, Zhaoxu Chen, Minchao Fang, Jie Yu, Cheng Zhu, Xianghan Zhang, Zhongliang Wang

**Affiliations:** ^1^ Lab of Molecular Imaging and Translational Medicine (MITM) Engineering Research Center of Molecular & Neuroimaging Ministry of Education School of Life Science and Technology Xidian University & International Joint Research Center for Advanced Medical Imaging and Intelligent Diagnosis and Treatment Xi'an Shaanxi 710126 P. R. China; ^2^ Guangzhou Institute of Technology Xidian University Guangzhou Guangdong 510555 P. R. China; ^3^ Tianjin Key Laboratory of Function and Application of Biological Macromolecular Structures School of Life Sciences Tianjin University 92 Weijin Road, Nankai District Tianjin 300072 P. R. China; ^4^ School of Biology and Engineering Guizhou Medical University Guizhou Guiyang 550025 P. R. China

**Keywords:** cancer therapy, filamentous actins, fusion, liposomes, protein delivery

## Abstract

Despite the potential of protein therapeutics, the cytosolic delivery of proteins with high efficiency and bioactivity remains a significant challenge owing to exocytosis and lysosomal degradation after endocytosis. Therefore, it is important to develop a safe and efficient strategy to bypass endocytosis. Inspired by the extraordinary capability of filamentous‐actin (F‐actin) to promote cell membrane fusion, a cyanine dye assembly‐containing nanoplatform mimicking the structure of natural F‐actin is developed. The nanoplatform exhibits fast membrane fusion to cell membrane mimics and thus enters live cells through membrane fusion and bypasses endocytosis. Moreover, it is found to efficiently deliver protein cargos into live cells and quickly release them into the cytosol, leading to high protein cargo transfection efficiency and bioactivity. The nanoplatform also results in the superior inhibition of tumor cells when loaded with anti‐tumor proteins. These results demonstrate that this fusogenic nanoplatform can be valuable for cytosolic protein delivery and tumor treatment.

## Graphical abstract

A cyanine dye assembly‐containing nanoplatform is first designed by mimicking the structure of natural filamentous‐actin, which can promote membrane fusion through increasing membrane tension. The nanoplatform displays superior cytosolic protein delivery through distinctive fusion toward the cell membrane. The high protein transfection and bioactivity indicate that the nanoplatform is feasible to improve intracellular protein delivery.

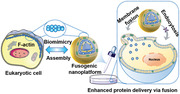

## 1 Introduction

Proteins play a significant role in almost every field of medicine^[^ ^1^ ^]^ owing to their characteristics of higher potency, specificity, and safety, in addition to being associated with faster clinical development, compared to small molecules.^[^ ^1b^ ^]^ However, because proteins are hydrophilic and macromolecular, it is difficult for them to cross the cell membrane, leading to current protein therapeutics mostly functioning against extracellular targets. One of the key issues to fully realize the therapeutic potential of proteins in cells is the delivery of active proteins to the cytosol.^[^ ^2^ ^]^ Currently, membrane‐permeable nanocarriers, such as liposomes,^[^ ^3^ ^]^ lipid nanoparticles,^[^ ^4^ ^]^ polymers,^[^ ^5^ ^]^ and inorganic nanoparticles^[^ ^6^ ^]^ can increase the specific cellular uptake of proteins; however, only a small fraction (<10%) of proteins reach the cytosol^[^ ^7^ ^]^ due to exocytosis^[^ ^8^ ^]^ and endocytic sequestration. Moreover, the bioactivity of proteins can be compromised by endosomal–lysosomal degradation. Although some strategies for facilitating endosomal escape have been developed, such as the acid‐responsive release of cargo^[^ ^9^ ^]^ and lysosome destruction by the proton sponge effect,^[^ ^10^ ^]^ their efficiency remains quite limited. Recently, researchers have proposed the concept of protein modification (cell‐penetrating peptide conjugation,^[^ ^11^ ^]^ bioreversible esterification,^[^ ^12^ ^]^ and cholesterol tagging^[^ ^13^ ^]^) to improve cytosolic protein delivery, whereby proteins can spontaneously cross the cell membrane and evade lysosomal degradation. However, these strategies typically require chemical synthesis, which might decrease protein bioactivity. Therefore, new delivery systems that not only bypass lysosomal degradation, but also are able to maintain protein bioactivity are critically important for advancing cytosolic protein delivery.

Membrane fusion^[^ ^14^ ^]^ provides one of the best solutions for delivering proteins into the cytosol with high efficiency and bioactivity, as fusogenic liposomes can, theoretically, directly fuse with the cell membrane and bypass lysosomal degradation. Over the past few years, several approaches, such as the incorporation of aromatic phospholipids,^[^ ^15^ ^]^ fusogenic peptides,^[^ ^16^ ^]^ and fusogenic lipids,^[^ ^17^ ^]^ have been developed to construct fusogenic liposomes. However, the cellular uptake of liposomes can be accompanied by endocytosis^[^ ^16^ ^]^ because this fusion is not fast enough to stop competitive endocytosis. To design fusogenic liposomes with high potency, we must learn from nature. Filamentous actin (F‐actin) polymerization^[^ ^18^ ^]^ promotes membrane fusion by increasing cell membrane tension^[^ ^19^ ^]^ and expanding membrane fusion pores.^[^ ^20^ ^]^ Given the typical size and complexity of native fusion proteins, simplified nanosystems capable of membrane fusion are required for cytosolic protein delivery.

**Scheme 1:**
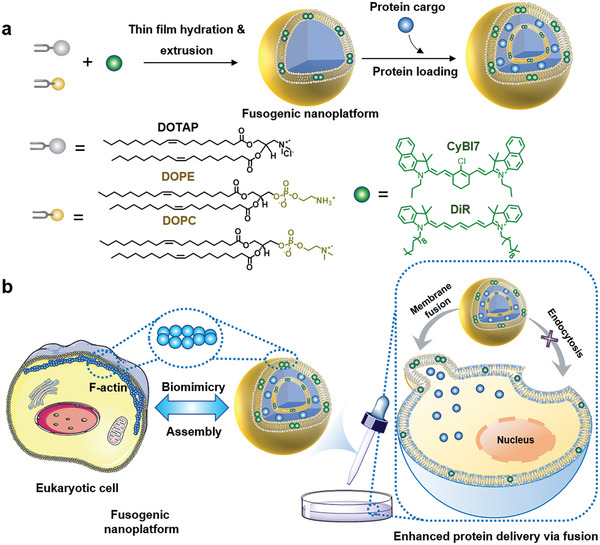
a) Fabrication of fusogenic nanoplatform and chemical structures of the components. Proteins distributed in the junctions between lipid bilayers. b) F‐actin‐mimicking strategy for enhanced cytosolic protein delivery via membrane fusion.

To build this type of fusogenic nanoplatform, we used a cationic liposome,^[^ ^21^ ^]^ a gene vector well‐studied for decades and currently under clinical trial,^[^ ^22^ ^]^ as the backbone because it not only loads proteins conveniently via simple mixing^[^ ^23^ ^]^ but also shows high affinity to cells through electrostatic attraction (**Scheme** **1**a). Near infrared (NIR) cyanine dyes were chosen to mimic F‐actin because they are not only easy to synthesize, wavelength‐tunable, and biocompatible but also can form assemblies in the lipid bilayer through *π*–*π* interactions^[^ ^24^ ^]^ and the presence of an assembly might greatly affect the fusion efficiency of the nanoplatform (Scheme 1b). Based on these designs, the nanoplatform can fuse with the cell membrane very quickly, and competing endocytosis is completely inhibited, resulting in highly efficient cytosolic protein delivery. We chose FITC (fluorescein isothiocyanate)‐labeled bovine serum albumin and enhanced green fluorescent protein as the protein cargos to visualize their cytosolic access, *β*‐galactosidase to verify the protein bioactivity and cellular uptake mechanism, and deoxyribonuclease I (DNase I) to test its anti‐tumor efficacy. This study thus provides the generation of a potent cytosolic protein delivery nanoplatform and proposes a versatile protein delivery paradigm using biomimetic strategies.

## 2 Results and Discussion

### 2.1 Design of F‐Actin‐Mimicking Liposomes

Two hydrophobic cyanine dyes with different assembly potentials in the lipid bilayer, CyBI7 and DiR (1,1′‐dioctadecyl‐3,3,3′,3′‐tetramethylindotricarbocyanine iodide, Scheme 1a), were chosen to mimic F‐actin. Rigid CyBI7 tends to form *π*–*π* assemblies in the lipid bilayer,^[^ ^25^ ^]^ whereas DiR, with two alkyl tails, is usually well‐dispersed in the lipid bilayer.^[^ ^26^ ^]^ To maximize the fusion potential of the nanoplatform, an equimolar mixture of 1,2‐dioleoyl‐3‐trimethylammonium‐propane (DOTAP) and 1,2‐dioleoyl‐sn‐glycero‐3‐phosphatidyl‐ethanolamine (DOPE) was chosen as the scaffold (named IL). These were selected because cationic DOTAP can increase the affinity toward cells through electrostatic attraction and DOPE can form an inverted hexagonal phase and stabilize the intermediate structures during membrane fusion.^[^ ^17^, ^27^ ^]^ To estimate the role of IL in the nanoplatform, an equimolar mixture of DOTAP and 1,2‐dioleoyl‐sn‐glycero‐3‐phosphocholine (DOPC) was used as the control scaffold (CL), where DOPC was column‐shaped and could not stabilize the fusion intermediates.

**Figure 1:**
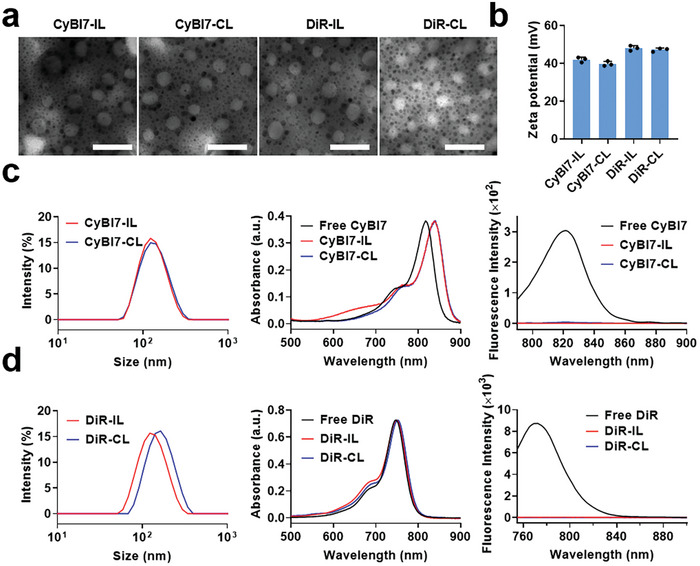
Characterization of the liposomes. a) Transmission electron microscopy (TEM) images of the liposomes. Scale bar, 100 nm. b) Zeta potentials of the cyanine dye‐containing liposomes. c) Size distributions, near infrared (NIR) absorption spectra, and fluorescence spectra of CyBI7‐containing liposomes. d) Size distributions, NIR absorption spectra, and fluorescence spectra of 1,1′‐dioctadecyl‐3,3,3′,3′‐tetramethylindotricarbocyanine iodide (DiR)‐containing liposomes. IL, 1,2‐dioleoyl‐3‐trimethylammonium‐propane and 1,2‐dioleoyl‐sn‐glycero‐3‐phosphatidyl‐ethanolamine scaffold; CL, control scaffold.

Four cyanine dye‐containing liposomes, CyBI7‐IL, CyBI7‐CL, DiR‐IL, and DiR‐CL were prepared by performing the thin‐film hydration method, and they showed a spherical morphology with average diameters of 118–135 nm and positive surface zeta potentials of +40–+48 mV (**Figure** **1**a,b). The highly positive zeta potential allowed the liposomes to have better structural stability in aqueous solutions (Figure S1, Supporting Information). The encapsulation efficiency and loading content of cyanine dyes in CyBI7‐IL, CyBI7‐CL, DiR‐IL, and DiR‐CL were 47.8%/2.4%, 50.0%/2.5%, 43.9%/2.2%, and 61.9%/3.1%, respectively (Figure S2a,b, Supporting Information). The ability of the cyanine dyes to self‐assemble in the liposome scaffolds was first evaluated based on NIR absorption and fluorescence emission spectra (Figure 1c,d). For CyBI7‐contained liposomes, the maximal absorption of CyBI7‐IL and CyBI7‐CL was 839 nm, which was bathochromically shifted by 22 nm compared with that of the CyBI7 monomer, suggesting the presence of *π*–*π* interaction based CyBI7 J‐aggregates.^[^ ^28^ ^]^ The fluorescence of CyBI7 was completely quenched (≈100%) in CyBI7‐IL and CyBI7‐CL because of the short intermolecular distance of CyBI7 in the assemblies. In addition, to evaluate the content of CyBI7 in lipid bilayer on the assembly, we also synthesized 1% and 3% CyBI7‐IL respectively. It was found that less CyBI7 did not affect the size and zeta potential (Figure S2c,d, Supporting Information), but the redshift was only observed in 3% CyBI7‐IL, indicating that sufficient CyBI7 is necessary for formation of J‐aggregation. For DiR‐containing liposomes, although the fluorescence of DiR was also completely quenched, a maximal absorption similar to that of the DiR monomer indicated that no DiR J‐aggregates were formed, which was probably due to interruption of *π*–*π* interaction by the long alkyl chain. As J‐aggregates are generally formed by both *π*–*π* interaction between polarized atoms and electrostatic interaction between opposite charges, we evaluated the role of electrostatic interaction in this assembly by synthesizing an ICG‐IL loading a negatively charged cyanine dye ICG, which is different from cationic CyBI7 or DiR. The maximum NIR absorption of ICG‐IL showed similar bathochromical shift to that of CyBI7‐IL (Figure S5c, Supporting Information), indirectly demonstrating that the major driving force for CyBI7 assembly in lipid bilayer is *π*–*π* interaction. Finally, to demonstrate that the dye‐containing liposomes could release dyes and restore fluorescence upon cellular uptake for further cell imaging studies, we chose the surfactant Triton X‐100 to disrupt liposome integrity, and the fluorescence was restored as expected (Figure S3, Supporting Information).

**Figure 2:**
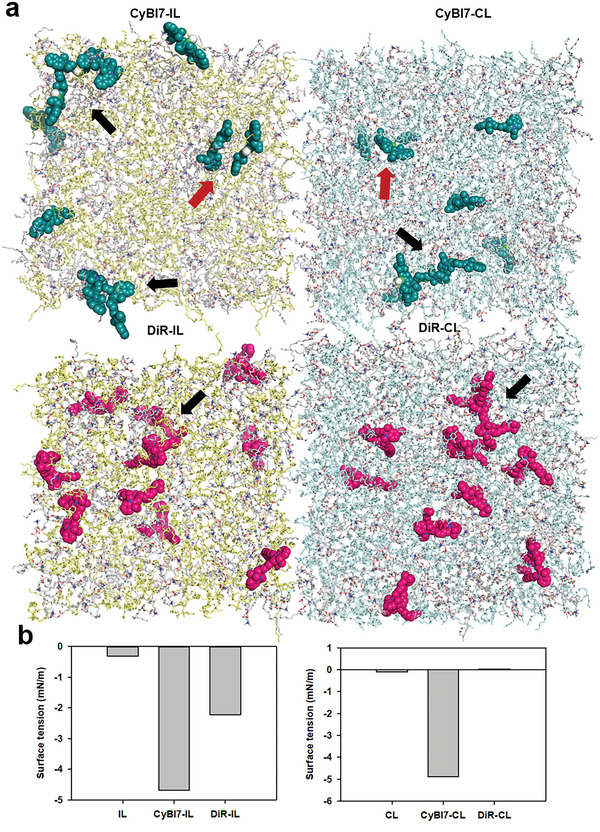
Molecular dynamic (MD) simulation of the liposomes. a) Representative configurations of MD simulations of *π* molecules in 2‐dioleoyl‐3‐trimethylammonium‐propane/1,2‐dioleoyl‐sn‐glycero‐3‐phosphatidyl‐ethanolamine (DOTAP/DOPE) and DOTAP/DOPC (1,2‐dioleoyl‐sn‐glycero‐3‐phosphocholine) lipid bilayers. The *π* molecules are depicted as large spheres, and phospholipids are represented as chains. The molecules are colored as follows: CyBI7, teal; 1,1′‐dioctadecyl‐3,3,3′,3′‐tetramethylindotricarbocyanine iodide (DiR), red; DOTAP, grey; DOPE, yellow; DOPC, cyan. For clarity, water molecules are not shown. Black arrows indicate the clusters. Red arrows indicate the typical J aggregates. b) Surface tension values of different liposomes.

To gain insight into the dispersed state of cyanine dyes in liposome scaffolds, we simulated the distribution of cyanine dyes in lipid bilayers using molecular dynamics. The interactions between the molecules CyBI7 or DiR in each system and the lipid bilayers (10 nm × 10 nm, DOTAP/DOPE 1/1, or DOTAP/DOPC 1/1) were simulated over 100 ns (**Figure** **2**a). For CyBI7‐containing formulations, at equilibrium, seven or eight of the 10 CyBI7 molecules formed small clusters (two to four molecules adjacent to each other and some exhibited “shifted plates” arrangement of typical J aggregates) in CyBI7‐IL and CyBI7‐CL, respectively. For DiR‐containing liposomes, although two and three of ten DiR molecules formed clusters, yet no typical J‐aggregate arrangement was observed. These calculations suggest that CyBI7 forms more ordered assemblies than DiR in the lipid bilayer.

To assess whether the assembled cyanine dyes can mimic F‐actin in its interaction with the membrane, we next calculated the surface tension of the four liposomes using molecular dynamics. F‐actin interacts with the cell membrane through an increase in the membrane tension, which then expands the membrane, enlarges the fusion pore, and promotes membrane fusion.^[^ ^20^ ^]^ According to the calculation, the surface tensions of IL/CyBI7‐IL/DiR‐IL and CL/CyBI7‐CL/DiR‐CL were −0.32/−4.7/−2.2 and −0.11/−4.9/0.04 mN m^−1^, respectively (Figure 2b). Negative surface tension implied that the membrane was compressed^[^ ^29^ ^]^ and tended to increase its surface area or expand the membrane, similar to the role of F‐actin in promoting membrane fusion. For a 10 µm radius liposome, the contribution to the tension from a thick filament (composed of F‐actin) is ≈10^−7^ N m^−1^.^[^ ^30^ ^]^ In our case, the change in membrane tension induced by CyBI7 dye assemblies is 4.7 mN m^−1^, equivalent to that by 4.7 × 10^4^ filaments. It is worth noting that the surface tension difference is enough to initiate the membrane change, as the lower bound for lysis tension of liposomes is 0.14 mN m^−1^.^[^ ^30^ ^]^ For ILs, both CyBI7‐IL and DiR‐IL had more negative surface tension, with the former being even more negative, indicating that both CyBI7 and DiR assemblies disturbed the membrane and increased membrane tension. Among the CLs, CyBI7‐CL still had a more negative surface tension, whereas DiR‐CL showed a positive surface tension. These results indicated that the abundant and ordered assemblies of CyBI7 could also disturb the membrane tension of the CL but that the scarce assemblies of DiR could not. Overall, these results suggest that CyBI7‐IL, CyBI7‐CL, and DiR‐IL mimic the interaction between F‐actin and the cell membrane to promote membrane fusion.

### 2.2 Membrane Fusion Efficiency of F‐Actin‐Mimicking Liposomes

**Figure 3:**
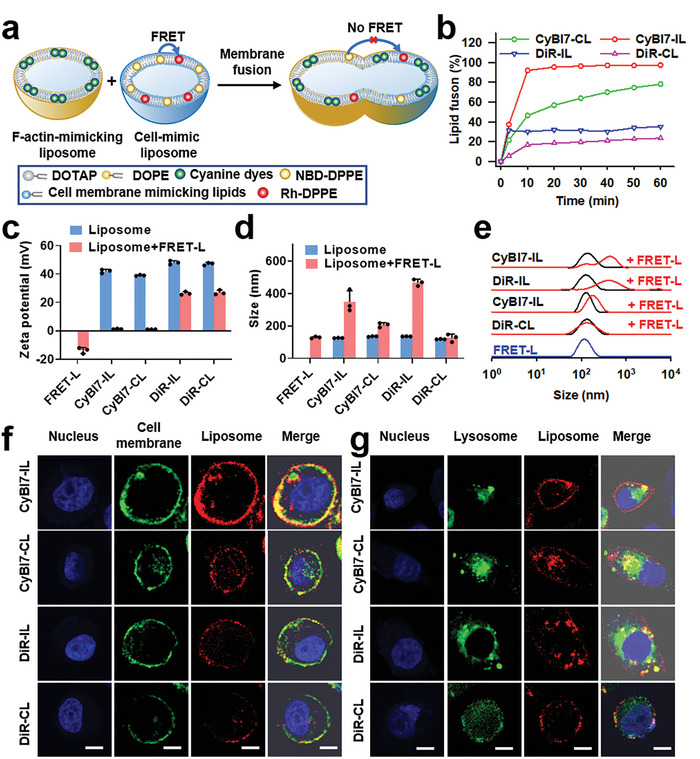
Membrane fusion characterization of F‐actin‐mimicking liposomes. a) Schematic illustration of the fusion process between F‐actin‐mimicking liposomes and FRET‐Ls (mimicking the cell membrane). b) Normalized lipid fusion kinetics of the liposomes in NaH_2_PO_4_ buffer (pH 7.4). c) Changes in the zeta potential, d) average size value, and e) size distribution of the liposomes before and after mixing with FRET‐L. f) Confocal images of the liposomes and cell membrane or g) lysosomes in BGC‐823 cells incubated with the liposomes for 15 min. Scale bar, 20 µm.

To determine whether F‐actin‐mimicking liposomes could promote membrane fusion, the fusogenic potential of the four liposomes toward cell membrane‐mimicking liposomes was quantified through Förster resonance energy transfer (FRET). FRET‐L, an anionic liposome containing DOPC and cholesterol at a 10/1 molar ratio (129 nm, −13.9 mV), as well as 1% NBD‐PE (donor) and 0.5% Rho‐PE (acceptor),^[^ ^31^ ^]^ was used as the cell membrane‐mimicking liposome, which showed a strong FRET effect. Once FRET‐L was fused with F‐actin‐mimicking liposomes, the FRET effect between NBD‐PE and Rho‐PE decreased. Therefore, we quantified the fusion efficiency between the liposomes by calculating the decrease in the FRET effect (the fluorescence intensity ratio of the acceptor to the donor; **Figure** **3**a; Figure S4, Supporting Information). Upon incubation with FRET‐L for 10 min, that of CyBI7‐IL, CyBI7‐CL, DiR‐IL, and DiR‐CL reached 91.9, 46.4, 30.2, and 16.9%, respectively (Figure 3b). Higher fusion potentials of CyBI7‐IL and CyBI7‐CL compared to those in the other groups were further confirmed based on the lower zeta potential after incubation (≈0 mV; Figure 3c). The steeper slopes in the fusion kinetic curves of the IL formulations, than those in the CL formulations, indicated that fusion occurred faster in the IL groups. Moreover, faster fusion might have led to larger sizes and wider distributions in the IL groups (Figure 3d,e). In addition, the IL exhibited a low fusion potential (5.4%, 20 min) and the ICG‐IL containing ICG assemblies in lipid bilayer demonstrated a high membrane fusion potential (55.2%, 20 min), highlighting the importance of F‐actin mimics in liposomal formulations for membrane fusion (Figure S5, Supporting Information). These results demonstrate that F‐actin‐mimicking cyanine aggregates can increase the fusion potential, whereas inverted‐shaped lipids can accelerate the membrane fusion process, resulting in the fastest fusion and the highest fusion efficiency of CyBI7‐IL toward cell membrane mimics.

To further evaluate the fusion of F‐actin‐mimicking liposomes with mammalian cells, we tracked cellular uptake of the liposomes. The four liposomes were readily taken up by BGC‐823 human gastric cancer cells within 1 h. CyBI7‐IL showed the strongest fluorescence and reached its maximum uptake as early as 30 min after incubation (Figure S6, Supporting Information). Meanwhile, the highest cellular uptake of CyBI7‐IL was observed in 4T1 murine breast cancer cells, MCF‐10A human epithelial cells, and Raw 264.7 murine macrophage cells (Figure S7, Supporting Information). To visualize the intracellular localization of liposomes, the membranes of BGC‐823 cells were stained with 3,3′‐dioctadecylloxacarbocyanine perchlorate (DiO, green fluorescence) or the lysosomes were stained with LysoTracker Green before imaging.^[^ ^16^, ^32^ ^]^ Liposomes were incubated with the cells for 15 min and immediately imaged using a confocal laser scanning microscope. CyBI7‐IL was found exclusively in the cell membrane, whereas the other liposomes (DiR‐IL, CyBI7‐CL, and DiR‐CL) were distributed in both the cell membrane and lysosomes (Figure 3f,g; Figure S9, Supporting Information). In addition, the colocalization images of the groups at 1 h showed similar results and confirmed the intracellular distributions in the different groups (Figures S8 and S9, Supporting Information). These results demonstrated that F‐actin‐mimicking liposomes can enter cells through membrane fusion.

Toxicity is a concern for the potent intracellular delivery of agents. We used the CCK‐8 assay to measure the cell viability of BGC‐823, 4T1, and MCF‐10A cells treated with 40–160 µm of liposomes for 24 h. All liposomes showed negligible toxicity, with cell viability greater than 80% with 160 µm, indicating the excellent biosafety of the liposomes (Figure S10a–c, Supporting Information). In addition, free CyBI7 exhibited excellent biosafety, with cell viability of greater than 90% with 20 µm (Figure S10d, Supporting Information). The favorable uptake mechanism and low toxicity of CyBI7‐IL led us to investigate its potential to transport protein cargo into cells.

### 2.3 Cytosolic Protein Delivery by F‐Actin‐Mimicking Liposomes

**Figure 4:**
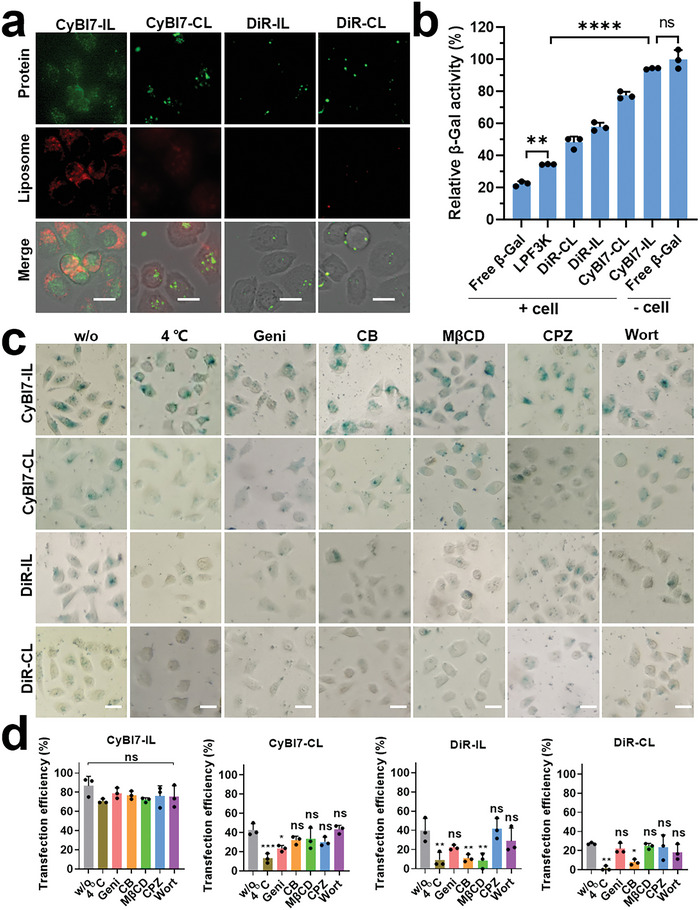
In vitro protein delivery with CyBI7‐IL. a) Fluorescence micrographs of BGC‐823 cells treated with liposome/BSA‐FITC complexes for 1 h. Scale bar, 50 µm. b) Quantitative *β*‐gal activity delivered by different carriers. c) Optical micrographs of BGC‐823 cells treated by liposome/*β*‐gal complexes under different conditions. w/o: without endocytosis inhibitor; Geni: genistein, caveolin‐mediated endocytosis inhibitor; CB: cytochalasin B, phagocytosis inhibitor; M*β*CD: methyl‐*β*‐cyclodextrin, lipid raft‐dependent endocytosis; CPZ: chlorpromazine, clathrin‐mediated endocytosis inhibitor; Wort: wortmannin, macropinocytosis inhibitor. Scale bar, 50 µm. d) Quantitative transfection efficiency of *β*‐gal in BGC‐823 cells after different treatments. Statistical significance in (b) and (d) was calculated via one‐way ANOVA with a Tukey test. ns, non‐significant, ^*^ *p* < 0.05, ^**^ *p* < 0.01, ^***^ *p* < 0.001, ^****^ *p* < 0.0001.

Membrane fusion‐mediated cellular uptake comprises a preferred method of protein delivery,^[^ ^15^ ^]^ which frees proteins from degradation in lysosomes. We first investigated whether liposomes could load cargo proteins, specifically FITC‐labeled bovine serum albumin (BSA‐FITC) and *β*‐galactosidase (*β*‐Gal), through electrostatic attraction. Dynamic light scattering results revealed that all liposome/protein complexes were positively charged (25–35 mV), and the average size was in the range of 150–350 nm (Figures S11 and S12, Supporting Information). The encapsulation efficiency of *β*‐Gal by the liposomes was ≈100%, as unencapsulated proteins were invisible on a native‐PAGE gel (Figure S12g, Supporting Information). The treatment of BGC‐823 cells with 20 µg mL^−1^ BSA‐FITC and 88 µm liposomes for 1 h resulted in the successful delivery of BSA‐FITC into the cytosol in a typical experiment (**Figure** **4**a). It has been reported that fluorescent proteins entrapped in endosomes are visible as highly localized and punctuated signals, whereas those free to move about the cytosol display diffuse and evenly spread signals.^[^ ^33^ ^]^ Therefore, the diffusely distributed fluorescence in the CyBI7‐IL group indicated the dispersion of BSA‐FITC in the cytosol, whereas the punctuated fluorescence in the other groups suggested that the proteins were partly entrapped in the lysosomes.

Next, we investigated whether the active proteins could be delivered into the cytosol. *β*‐Gal was chosen as the protein cargo because of its ability to catalyze the conversion of colorless X‐Gal (5‐bromo‐4‐chloro‐3‐indolyl‐*β*‐D‐galactoside) into a blue product^[^ ^9^, ^34^ ^]^ (Figure S13a, Supporting Information). To evaluate protein delivery efficiency, free *β*‐Gal and its complex with commercial Lipofectamine 3000 (LPF3K) were used as negative and positive controls, respectively. As shown in Figure S13b (Supporting Information), the treatment of BGC‐823 cells with 6 µg mL^−1^ *β*‐Gal and liposomes (48 µm) or LPF3K for 1 h resulted in the successful delivery of *β*‐Gal into the cytosol, where they all showed obvious blue precipitates. In comparison, free *β*‐Gal could barely enter cytosol, leading to negligible blue precipitate contents. Further quantification of the *β*‐Gal transfection efficiency^[^ ^35^ ^]^ revealed that the CyBI7‐IL group achieved the highest value (89%) after only 1 h of incubation, which was much higher than that in the other groups, specifically, 39% (CyBI7‐CL), 49% (DiR‐IL), 32% (DiR‐CL), and 4.7% (LPF3K) (Figure S13b,c, Supporting Information). Enzymatic bioactivity comprises a particularly stringent test for bioactivity retention after transfection, which is a key issue in protein delivery.^[^ ^36^ ^]^ Enzyme activity refers to the remaining enzyme activity after cellular uptake of liposome/protein complexes compared to free enzyme without any pretreatment. To measure the enzymatic activity of *β*‐Gal in the cells, BGC‐823 cells were treated with free *β*‐Gal, liposome/*β*‐Gal complexes, or LPF3K/*β*‐Gal complexes (*β*‐Gal: 6 µg mL^−1^) for 1 h and then lysed for the quantification of enzymatic activity. As shown in Figure 4b, the enzyme activity in the CyBI7‐IL group was the highest (94%), as compared to values of 22–78% obtained in the other groups (CyBI7‐CL: 78%; DiR‐IL: 58%; DiR‐CL: 48%; LPF3K: 34%; free *β*‐Gal: 22%). In addition, CyBI7‐IL could deliver *β*‐Gal to ≈100% 4T1 and ≈50% MCF‐10A cells within 1 h (Figure S14, Supporting Information), implying the universality of CyBI7‐IL in protein cytosol delivery. These results demonstrate that F‐actin‐mimicking liposomes can improve the protein subcellular distribution, enhance protein transfection efficiency, and elevate protein bioactivity.

Given the high protein transfection efficiency and high enzymatic activity facilitated by F‐actin‐mimicking liposomes, we sought to test the cellular uptake pathways of the liposome/*β*‐Gal complexes in BGC‐823 cells. For this, the cells were treated with various endocytic inhibitors^[^ ^37^ ^]^ before incubation with the complexes (Figure 4b,c). Generally, a 4 °C treatment can block endocytosis^[^ ^13^ ^]^ and is used to estimate the role of endocytosis in cellular uptake. After the 4 °C treatment, the transfection efficiency in the CyBI7‐IL group was only reduced by 18%, whereas that in the CyBI7‐CL, DiR‐IL, and DiR‐CL groups was substantially reduced by 70%, 76%, and 95%, respectively. This indicates that CyBI7‐IL entered cells by bypassing energy‐dependent endocytosis, whereas the other compounds entered cells partly through endocytosis. As shown in Figure 4c,d, further inhibitor treatments revealed the detailed mechanisms underlying endocytosis pathways; specifically, CyBI7‐CL entered cells through caveolin‐mediated endocytosis, DiR‐IL entered cells through phagocytosis and lipid raft‐dependent endocytosis, and DiR‐CL entered cells through phagocytosis. For cellular uptake, membrane fusion and endocytosis compete. For CyBI7‐IL, it exhibits quite fast fusion and reached >95% membrane fusion in 10 min, and therefore the membrane fusion dominates and surpasses endocytosis. For CyBI7‐CL, although it also exhibits high fusion at 1 h (≈80%), yet the fusion is rather slow and the competitive endocytosis occurs at the same time, leading to much lower transfection efficiency (≈40%). These results emphasize the importance of IL in the CyBI7‐IL formulation, where the inverted‐cone shaped DOPE accelerates membrane fusion through stabilizing the fusion intermediates.^[^ ^17^, ^27^ ^]^ These experiments demonstrate that the higher protein transfection and enzymatic activity in the CyBI7‐IL group was due to a distinct cellular uptake pathway, namely, complete membrane fusion and the absence of endocytosis.

**Figure 5:**
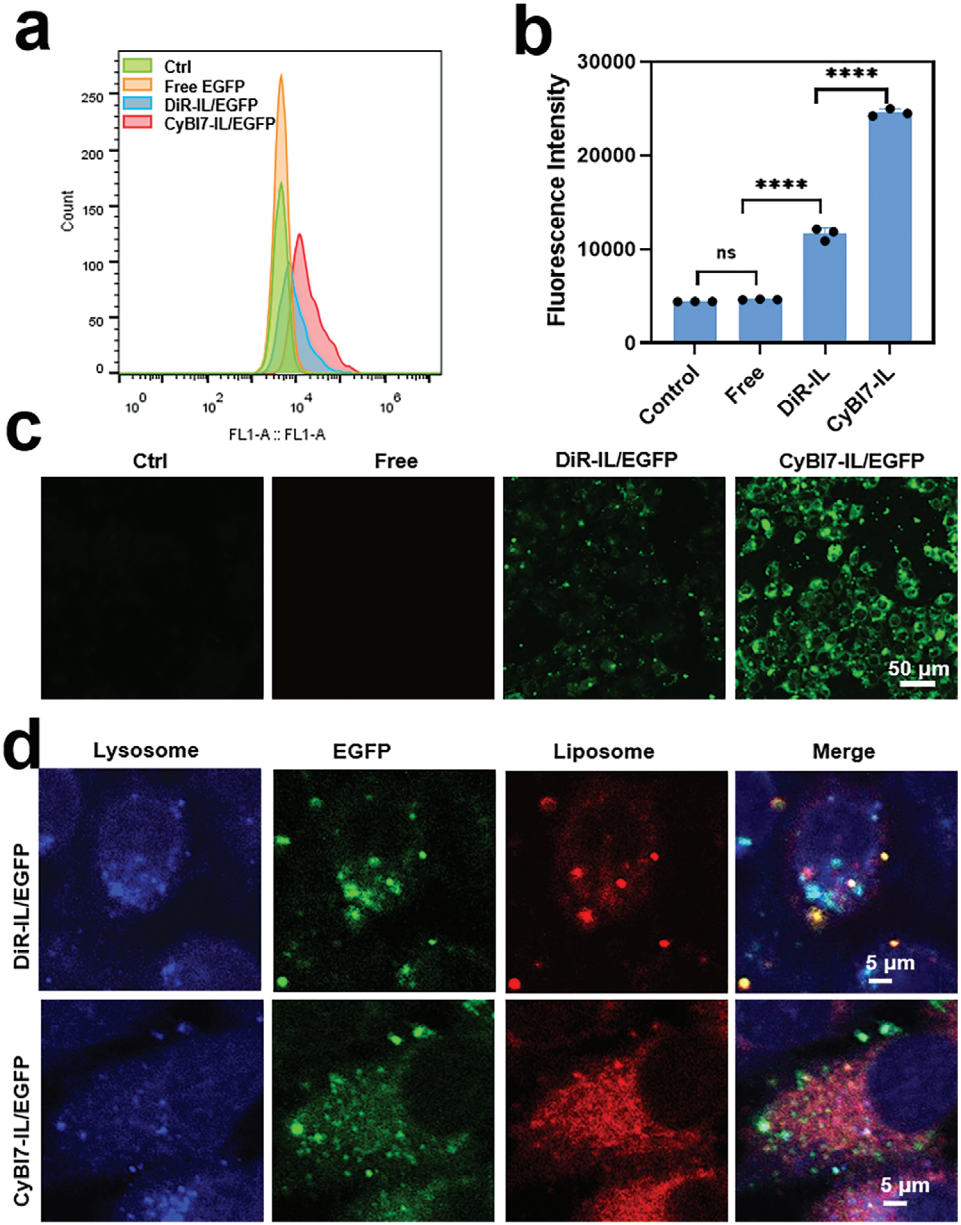
In vitro EGFP delivery with CyBI7‐IL. a) Flow cytometric histograms of BGC‐823 cells incubated with different EGFP‐contained formulations for 1 h. b) Quantification of mean fluorescence intensity (MFI) of the treated cells in (a). Data are shown as mean ± SD (n = 3). c) Fluorescence micrographs of BGC‐823 cells treated with EGFP‐contained formulations for 1 h. Scale bar, 50 µm. d) Confocal images of the liposomes and lysosomes in BGC‐823 cells incubated with the liposome/EGFP complexes for 1 h. Scale bar, 5 µm.

To further demonstrate the in vitro protein delivery efficacy with our liposomes, we chose fluorescent enhanced green fluorescent protein (EGFP, pKa = 6.2, MW = 26.9 kDa) as another model protein. Since the EGFP carries very weak charge in neutral pH, we optimized the pH for preparing CyBI7‐IL/EGFP complexes for cytosolic EGFP delivery, which was pH = 8 (Figure S15, Supporting Information). Later, we prepared the liposome/EGFP complexes at the optimal condition and compared the EGFP transfection by the two liposome formulations (CyBI7‐IL and DiR‐IL) with flow cytometry. As shown in **Figure** **5**a,b, CyBI7‐IL transfected more EGFP than DiR‐IL, while free EGFP cannot enter cells by itself. Further fluorescence micrographs showed more cytosolic EGFP delivery in CyBI7‐IL group (Figure 5c). Further, lysosome colocalization images demonstrated that CyBI7‐IL/EGFP bypassed lysosomes but most of DiR‐IL/EGFP was entrapped in lysosomes (Figure 5d). In addition, we also evaluated the effect of CyBI7 content in CyBI7‐IL on protein transfection. As shown in Figure S16 (Supporting Information), liposomes with less dyes showed less protein delivery, demonstrating the key role of dye in cytosolic delivery. By delivering three model proteins (BSA‐FITC, *β*‐Gal, and EGFP), we demonstrated that CyBI7‐IL can deliver protein with high bioactivity bypassing endosomes.

**Figure 6:**
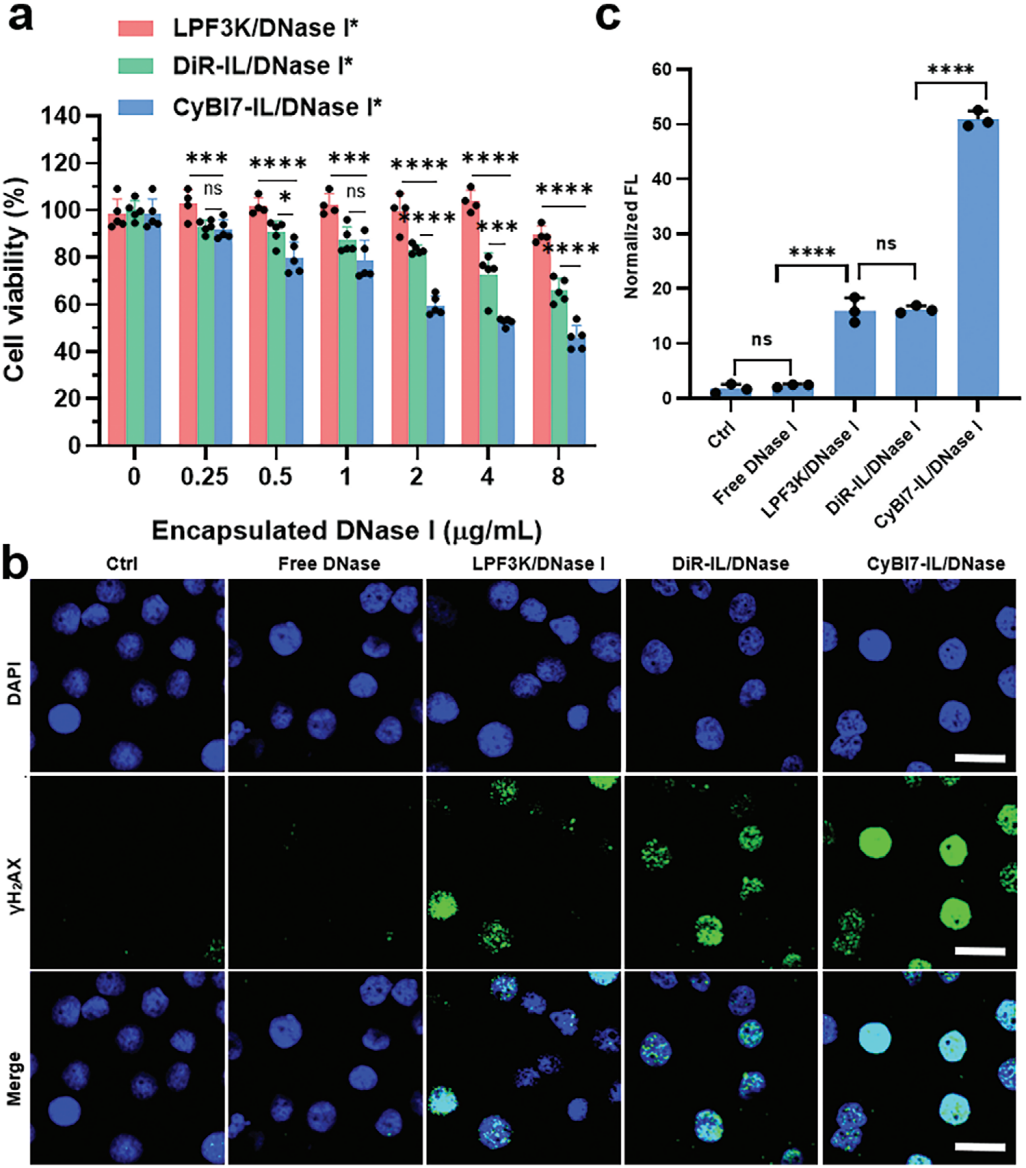
In vitro therapeutic efficacy of DNase I delivered by F‐actin‐mimicking liposomes. a) Cell viability of BGC‐823 cells after different treatments for 24 h. b) CLSM images of *γ*‐H2AX immunofluorescence staining of BGC‐823 cells after different treatments. Scale bar, 20 µm. c) Quantification of *γ*‐H2AX immunofluorescence in different groups. Statistical significance in (a) and (c) was calculated via one‐way ANOVA with a Tukey test. ns, non‐significant, ^*^ *p* < 0.05, ^**^ *p* < 0.01, ^***^ *p* < 0.001, ^****^ *p* < 0.0001.

Having demonstrated the superiority of F‐actin‐mimicking liposomes in cytosolic protein delivery, we investigated whether liposomes could improve the therapeutic efficacy of anti‐tumor proteins. DNase I, which digests DNA phosphodiester bonds and inhibits cell growth,^[^ ^38^ ^]^ was chosen as the anti‐tumor protein. The liposome/DNase I complexes were found to be 150–250 nm in size and were positively charged (25–40 mV) (Figure S17a,b, Supporting Information). The encapsulation efficiencies of DNase I by LPF3K, DiR‐IL, CyBI7‐IL were 32.3%, 68.0%, and 82.0%, respectively (Figure S17c,d, Supporting Information). The lower encapsulation efficiency of DNase I compared to that of *β*‐Gal was probably due to its weaker negative charge in neutral conditions (pI_DNase I_ = 5, pI*_β_*_‐gal_ = 4.6). To compare the DNase I delivery efficiencies by different formulations in the same encapsulated DNase I encapsulated concentration, 3.1‐fold LPF3K/DNase I complexes, 1.5‐fold LPF3K/DNase I complexes, and 1.2‐fold LPF3K/DNase I complexes were used to treat the cells, as their encapsulation efficiencies varied greatly. To distinguish them from other liposome/DNase I complexes with same total DNase I concentration, the formulations here were all marked with asterisk. As shown in **Figure** **6**a, CyBI7‐IL/DNase I^*^ showed the best therapeutic efficacy in all treated concentrations while the blank carriers and free DNase I at the highest concentration were nontoxic (CyBI7‐IL 109 µM, DiR‐IL 132 µM, LPF3K, 1:220 dilution, free DNase I 24 µg mL^−1^, Figure S10a and S17e, Supporting Information), demonstrating the excellent protein delivery efficiency of CyBI7‐IL. This increase in tumor cell inhibition was expected to be due to DNase I‐induced DNA damage. Therefore, we further investigated DNA damage in different groups by staining for the DNA double‐strand break‐induced biomarker phosphorhistone 2AX (*γ*‐H2AX).^[^ ^39^ ^]^ The CyBI7‐IL group showed the strongest green fluorescence in the cell nucleus, much stronger than that in the other liposomal groups (DiR‐IL and LPF3K groups), which also exhibited obvious green fluorescence in the cell nucleus. The free DNase I group showed negligible green fluorescence. The DNA damage results were consistent with the excellent therapeutic efficacy of CyBI7‐IL‐delivered DNase I. These results confirmed that F‐actin‐mimicking liposomes can improve the therapeutic outcome of anti‐tumor proteins through distinct membrane fusion events.

## 3 Conclusion

In summary, we demonstrated that a hydrophobic cyanine dye‐containing liposome, CyBI7‐IL, is a potent protein delivery vehicle. Our studies on CyBI7‐IL further showed that CyBI7 assembly in the lipid bilayer can indeed mimic F‐actin, which was found to increase the membrane tension and fusion potential in experiments and molecular simulations. Subsequently, we demonstrated that the F‐actin‐mimicking nanoplatform CyBI7‐IL could deliver proteins with a high transfection efficiency and bioactivity, as the transfection occurred through membrane fusion and was independent of endocytosis, bypassing lysosomal degradation. Finally, we verified that CyBI7‐IL can be used to deliver anticancer protein therapeutics and enhance tumor cell inhibition. This study provides a useful protein delivery tool that is well suited for a variety of applications. As this nanoplatform delivers proteins via electrostatic attraction, the F‐actin‐mimicking strategy presented here offers a direction for the creation of non‐viral gene carriers, holding long‐term potential for gene and cell therapies.

## 4 Experimental Section

### Materials

1,2‐dioleoyl‐sn‐glycero‐3‐phosphoethanolamine (DOPE), 1,2‐dioleoyl‐sn‐glycero‐3‐phosphocholine (DOPC), 1,2‐dioleoyl‐3‐trimethylammonium‐propane (chloride salt, DOTAP), 1,2‐dipalmitoyl‐sn‐glycero‐3‐phosphoethanolamine‐N‐(7‐nitro‐2‐1,3‐benzoxadiazol‐4‐yl) (ammonium salt, NBD‐PE) and 1,2‐dipalmitoyl‐sn‐glycero‐3‐phosphoethanolamine‐N‐(lissamine rhodamine B sulfonyl) (ammonium salt, Rho‐PE) were purchased from Avanti Polar Lipids; 1,1′‐dioctadecyl‐3,3,3′,3′‐tetramethylindotricarbocyanine iodide (DiR) was from AAT Bioquest; CyBI7 was synthesized following a reported protocol.^[^ ^25^ ^]^ *β*‐galactosidase, *β*‐galactosidase Assay Kit, In Situ *β* galactosidase Staining Kit and FITC labeled bovine serum albumin (BSA‐FITC) were from Solarbio life sciences; enhanced green fluorescent protein (EGFP) was purchased from KMI‐Kangma Intelligence. Deoxyribonuclease I (DNase I) from bovine pancreas (protein ≥ 85%, ≥ 400 kunitz mg^−1^ protein) were obtained from Sigma–Aldrich. Lipofectamine 3000 (LPF3K) was purchased from Invitrogen. Human gastric cancer cells (BGC‐823), murine breast cancer cells (4T1), and human breast endothelial cells (MCF‐10A) were obtained from the American Type Culture Collection (ATCC). RPMI‐1640, DMEM and fetal bovine serum (FBS) were purchased from Gibco; 4′,6‐diamidino‐2‐phenylindole (DAPI), trypsin and Penicillin‐Streptomycin (P/S, 10000 U mL^−1^) were purchased from HyClone; Cell Counting Kit‐8 (CCK‐8) was from Shanghai Yuanye Bio‐Technology Co., Ltd.; DiO (3,3′‐dioctadecyloxacarbocyanine perchlorate) was purchased from Beyotime Biotechnology. Lysosome tracker was purchased from Yeasen Biotechnology (Shanghai) Co., Ltd. LysoTracker Blue DND‐22 was purchased from Thermofisher. Deionized water (18.2 MΩ cm, 25 °C) was used in the study.

### Preparation and Characterization of Liposomes

The liposomes (CyBI7‐IL, DiR‐IL) were prepared with thin‐film hydration method. To prepare CyBI7‐IL, CyBI7 (1 µmol), DOPE (10 µmol), and DOTAP (10 µmol) dispersed in 3 mL of mixed organic solvent (chloroform/methanol, 9/1, v/v) was added into a pear‐shaped flask, and the organic solvent was removed by sequential rotary evaporation (15 min) and vacuum desiccation (overnight). Then the dry lipid membrane was hydrated with water (4 mL) to form multilamellar vesicles and the vesicles were further homogenized after extrusion (100 nm polycarbonate, ten times) under nitrogen. In the end, the homogenized liposomes were dialyzed against water to remove unencapsulated CyBI7. The liposomes DiR‐IL were prepared similarly to CyBI7‐IL, except that the added CyBI7 was replaced with DiR (1 µmol). The liposomes CyBI7‐CL and DiR‐CL were prepared similarly to CyBI7‐IL and DiR‐IL, except that the added DOPE was replaced with DOPC (10 µmol). The sizes and zeta potentials of the liposomes were measured with dynamic light scattering (Malvern ZEN3690). The transmission electron microscopy (TEM) imaging of liposomes was performed on HT7700 and the sample preparation was the same as the previous work.^[^ ^40^ ^]^ The encapsulation efficiencies of CyBI7 and DiR in the liposomes were determined according to the NIR absorbances of CyBI7 (819 nm) and DiR (748 nm) in disrupted liposomes with 0.05% Triton X‐100.^[^ ^41^ ^]^ The loading contents of dyes in the liposomes were the amount of total entrapped dyes divided by the total lipids (mol/mol). The fluorescence spectra of CyBI7 and DiR in the liposomes and disrupted liposomes (with 0.05% Triton X‐100) were measured with excitation at 780 and 745 nm, respectively.

### Molecular Dynamics Simulation

All‐atom MD simulations based on the GROMACS 2020.6 package^[^ ^42^ ^]^ were performed to investigate the interactions between *π* molecules and a simulated lipid bilayer. CGenFF^[^ ^43^ ^]^ was employed to generate the topologies and parameters of *π* molecules as well as DOTAP, DOPE, and DOPC, that were compatible with the CHARMM36 force field.^[^ ^44^ ^]^ The DOTAP/DOPE lipid bilayer was represented by a mixed lipid bilayer composed of 50% DOTAP, 50% DOPE (or DOPC), and 5% CyBI7 (or DiR) for a total of ≈220 lipid molecules or a total of 131 000–179 000 atoms (including waters and ions) for the whole system. The simulations started with ten of *π* molecules (CyBI7 or DiR) randomly distributed in the bilayer of 10 nm × 10 nm, and 100 ns production runs were performed after equilibrium for three independent trajectories,^[^ ^45^ ^]^ following previously reported procedures. Quantitative parameters, such as distances between *π* molecules and surface tension, were extracted using gmx distance and gmx energy. Water molecules were not shown for clarity.

### Fusogenic Efficiency of Liposomes

To investigate the fusogenic properties of the liposomes, FRET‐L (DOPC/Chol/NBD‐PE/Rho‐PE 10/1/0.1/0.05, mol/mol) were first prepared,^[^ ^31a^ ^]^ which mimics the composition of cell membrane. Then, the liposomes (CyBI7‐IL, DiR‐IL, CyBI7‐CL, DiR‐CL and IL 2 mm, 90 µL) were mixed with FRET‐L (2 mm, 10 µL) and diluted in phosphate buffer (10 mm, pH = 7.4, 500 µL) and the recovery of fluorescence energy transfer (FRET) between NBD‐PE and Rho‐PE (EX 475 nm, EM 500–700 nm) was tracked at different times (3, 13, 23, 33, 43, 53, and 63 min) to monitor the membrane fusion process. To quantify the FRET effect, the standard liposomes CyBI7‐F100L (DOPC/Chol/NBD‐PE/Rho‐PE/CyBI7 10/1/0.01/0.005/0.50), DiR‐F100L (DOPC/Chol/NBD‐PE/Rho‐PE/DiR 10/1/0.01/0.005/0.50) and F100L (DOPC/Chol/NBD‐PE/Rho‐PE 10/1/0.01/0.005) were further prepared. The CyBI7‐F100L mimics the composition of mixed liposomes after 100% fusion between FRET‐L and CyBI7‐contained liposomes. Similarly, the DiR‐F100L and F100L mimic the 100% fusion in the DiR‐contained formulation and IL, respectively. The fusion efficiency was calculated according to the following formula:

(1) $$\eta =\left(R_{t}-R_{0}\right)/\left(R_{100}-R_{0}\right)\;\times \;100\%$$

where *R*_t_ is the acceptor/donor fluorescence ratio (EX475 nm, EM 592 nm/EM 516 nm) of FRET‐L/liposome mixture at different times, *R*_0_ is the ratio of FRET‐L (2 mm, 10 µL) in phosphate buffer (590 µL) and *R*_100_ is the ratio of CyBI7‐F100L, DiR‐F100L or F100L (2 mm, 100 µL) in phosphate buffer (500 µL).

### Cellular Uptake of Liposomes

BGC‐823 cells were cultured in RPMI‐1640 media (10% FBS, 1% P/S) at 37  °C in 5% CO_2_ atmosphere. The cells were first seeded in 96‐well plate at a density of 0.5–1×10^4^ cells per well and incubated for 24 h. Later, the old cell media were removed, and the cells were treated with serum‐free media containing liposomes (CyBI7‐IL, DiR‐IL, CyBI7‐CL, DiR‐CL, 40 μM lipids, 100 µL per well) for different times (5, 10, 20, 30, and 60 min). Then the cells were washed with PBS and treated with fresh cell media before fluorescence imaging (Leica).

### Cell Membrane Colocalization

The BGC‐823 cells were first seeded in eight‐chamber confocal dish and when the cell confluency reached 80%, the old cell media were removed and the cells were treated with serum‐free media containing liposomes (CyBI7‐IL, DiR‐IL, CyBI7‐CL, DiR‐CL, 160 μm lipids, 400 µL per welll) for 15 and 60 min. Then, the cells were washed with PBS and treated with DiO contained cell media (1×, 200 µL) for 5 min. Later, the cells were fixed with paraformaldehyde solution (4%, 1 mL) for 25 min. Next, the cells were incubated with DAPI solution (4 µg mL^−1^, 20 µL) for 5 min and washed with PBS. Finally, the cells were treated with anti‐fading agent (100 µL) before confocal laser scanning imaging.

### Lysosome Colocalization

The BGC‐823 cells were first treated with liposomes similarly to that in the study of cell membrane colocalization. Then the cells were washed with PBS and stained with lysosome tracker (10 μM) for 30 min. Next, the cells were fixed and stained with DAPI similarly. Finally, the cells were treated with anti‐fading agent before confocal laser scanning imaging.

### Preparation of Liposome/BSA‐FITC Complexes

The complexes were prepared by mixing liposomes (2 mm, 44 µL) and BSA‐FITC (1 mg mL^−1^, 20 µL) for 5 min. The size distributions and zeta potentials of liposome/BSA‐FITC complexes were measured by dynamic light scattering.

### Fluorescence Imaging of BSA‐FITC

The BGC‐823 cells were seeded in 96‐well plates (5000 cells per well) and when the cell confluency reached 80%, the cells were treated with liposome/BSA‐FITC complexes (20 µg mL^−1^ BSA‐FITC) for 60 min. Then the cells were washed with PBS and observed under fluorescence microscopy (Leica).

### Preparation of Liposome/*β*‐Galactose Complexes

The complexes were prepared by mixing liposomes (2 mm, 40 µL) and *β*‐galactose (*β*‐Gal, 1 mg mL^−1^, 11 µL) for 5 min. The size distributions and zeta potentials of liposome/*β*‐Gal complexes were measured by dynamic light scattering.

### β‐Gal Staining In Situ

The BGC‐823 cells were seeded in 96‐well plates (5000 cells per well) and when the cell confluency reached 80%, the cells were treated with liposome/*β*‐Gal complexes, LPF3K/*β*‐Gal complexes and free *β*‐Gal (6 µg mL^−1^ *β*‐Gal) for 60 min. Then the cells were washed with PBS and stained according to the manufacturer's protocol. Finally, the intracellular distribution of *β*‐Gal was observed under bright field. The transfection efficiency of *β*‐Gal was determined by the percentage of positively stained cells.^[^ ^35^ ^]^ For counting, four pictures (each having ≈30 cells) were taken randomly from each replica and then averaged.

### *β*‐Gal Activity

To determine the β‐Gal activity after transfection, the BGC‐823 cells were first treated with different complexes (liposome/β‐Gal, LPF3K/β‐Gal) and free β‐Gal for 1 h and then treated with cell lysis buffer for 60 min. Later, the β‐Gal activity in lysed cells were determined by measuring the optical density at 420 nm after reacting with detection agent for 3 h at 37  °C according to the manufacturer's protocol. The related activity was the optical density of lysed samples compared to that of standard β‐Gal at the same concentrations.

### Preparation of Liposome/EGFP Complexes

The complexes were prepared by mixing liposomes (2 mm, 40 µL), phosphate buffer (10 mm, pH = 8, 4 µL), and EGFP (1 mg mL^−1^, 10 µL) and incubated for 15 min. The size distributions and zeta potentials of liposome/EGFP complexes were measured by dynamic light scattering.

### Fluorescence Imaging of Cells Treated by Liposome/EGFP Complexes

The BGC‐823 cells seeded in 96‐well plates were treated with liposome/EGFP complexes (10 ng µL^−1^ EGFP) for 60 min. Then the cells were washed with PBS and observed under fluorescence microscopy (Leica).

### Flow Cytometry of Cells Treated by Liposome/EGFP Complexes

The BGC‐823 cells seeded in 24‐well plates were treated with liposome/EGFP complexes (10 ng µL^−1^ EGFP) for 60 min. Then the cells were washed with PBS and collected for flow cytometry analysis (BD Accuri C6).

### EGFP and Lysosome Colocalization

The BGC‐823 cells were first treated with liposome/EGFP complexes (EGFP 10 µg mL^−1^) for 60 min. Then the cells were washed with PBS and stained with LysoTracker Blue DND‐22 (10 μm) for 30 min. Finally, the cells were treated with anti‐fading agent before confocal laser scanning imaging.

### Preparation of Liposome/DNase I Complexes

The complexes were prepared by mixing liposomes (2 mm, 11.2 µL) and DNase I (1 mg mL^−1^, 2 µL) for 15 min. The size distributions and zeta potentials of liposome/DNase I complexes were measured by dynamic light scattering.

### Cytotoxicity of Liposome/DNase I Complexes

The BGC‐823 cells were first treated with different liposome complexes (3.1‐fold LPF3K/DNase‐I (LPF3K, 1:220 dilutions, DNase I, 24 µg mL^−1^), 1.5‐fold DiR‐IL/DNase I (DiR‐IL 132 µM, DNase I 12.8 µg mL^−1^) and 1.2‐fold‐CyBI7‐IL/DNase I (CyBI7‐IL: 109 µM, DNase I 9.6 µg mL^−1^) containing same encapsulated DNase I (8 µg mL^−1^) in serum‐free medium for 1 h. For the other DNase I concentration treatment, the cells were treated with serial dilution method in the plates and incubated in serum‐free medium for 1 h. Then the cells were all cultured in serum‐containing medium for further 23 h. Later, the cell viability in different groups was measured by MTT assay.

### DNA Damage Assay

The BGC‐823 cells were seeded in eight well chamber slides at a density of 50 000 cells per well and incubated for further 12 h. When the cell confluency reached 60–70%, the cells were treated with liposome/DNase I complexes and free DNase I (DNase I, 2 µg mL^−1^) in serum‐free medium for 1 h. Then the cells were further cultured in fresh serum‐containing medium for 23 h. Finally, the cells were treated with *γ*‐H2AX immunostaining assay and observed under CLSM.

### Statistical Analysis

Statistical differences between the two groups were calculated via one‐way ANOVA with a Tukey test using GraphPad Prism 8.0 (GraphPad Software, Inc., CA, USA), and *p* < 0.05 was statistically significant. Asterisk (^*^) denotes statistical significance between bars (^*^ *p* < 0.05, ^**^ *p* < 0.01, ^***^ *p* < 0.001, ^****^ *p* < 0.001).

## Conflict of Interest

The authors declare no conflict of interest.

## Supporting information

Supporting Information

## Data Availability

The data that support the findings of this study are available from the corresponding author upon reasonable request.
