# Supplementary material for: Filamentous‐Actin‐Mimicking Nanoplatform for Enhanced Cytosolic Protein Delivery
Source: Adv Sci (Weinh). 2023 Dec 28;11(10):2305600. doi: 10.1002/advs.202305600 (PMC10933650; doi:10.1002/advs.202305600)
Supplement: Supplementary file 1 — Supporting Information [file ADVS-11-2305600-s001.pdf]

ADVANCED SCIENCE
Open Access

Supporting Information

for *Adv. Sci.*, DOI 10.1002/advs.202305600

Filamentous-Actin-Mimicking Nanoplatform for Enhanced Cytosolic Protein Delivery

Yuqiong Xia, Keyun Wu, Chang Liu, Xuejuan Zhao, Jun Wang, Jianxia Cao, Zhaoxu Chen, Minchao Fang, Jie Yu, Cheng Zhu, Xianghan Zhang and Zhongliang Wang**

Supporting Information

Filamentous-actin-mimicking Nanoplatform for Enhanced Cytosolic Protein Delivery

Yuqiong Xia[a][b], Keyun Wu[a][b], Chang Liu[a][b], Xuejuan Zhao[a][b], Jun Wang[a], Jianxia Cao[a], Zhaoxu Chen[a], Minchao Fang[c], Jie Yu[d], Cheng Zhu[c], Xianghan Zhang[a][b], and Zhongliang Wang*[a]*

[a] Prof. Y. Xia, K. Wu, C. Liu, X. Zhao, J. Wang, J. Cao, Z. Chen, Prof. X. Zhang, Prof. Z. Wang
Lab of Molecular Imaging and Translational Medicine (MITM), Engineering Research Center of Molecular & Neuroimaging, Ministry of Education, School of Life Science and Technology, Xidian University & International Joint Research Center for Advanced Medical Imaging and Intelligent Diagnosis and Treatment, Xi'an, Shaanxi, 710126, P. R. China
E-mail: xhzhang@xidian.edu.cn, wangzl@xidian.edu.cn
[b] Prof. Y. Xia, Keyun Wu, Chang Liu, Xuejuan Zhao, Prof. X. Zhang
Guangzhou Institute of Technology, Xidian University, Guangzhou, Guangdong, 510555, China.
[c] M. Chao, Prof. C. Zhu
Tianjin Key Laboratory of Function and Application of Biological Macromolecular Structures, School of Life Sciences, Tianjin University, 92 Weijin Road, Nankai District, Tianjin 300072, China.
[d] Dr. J. Yu
Immune Cells and Antibody Engineering Research Center of Guizhou Province, Key Laboratory of Biology and Medical Engineering, Guizhou Medical University, Guiyang, 550025, China

Figures

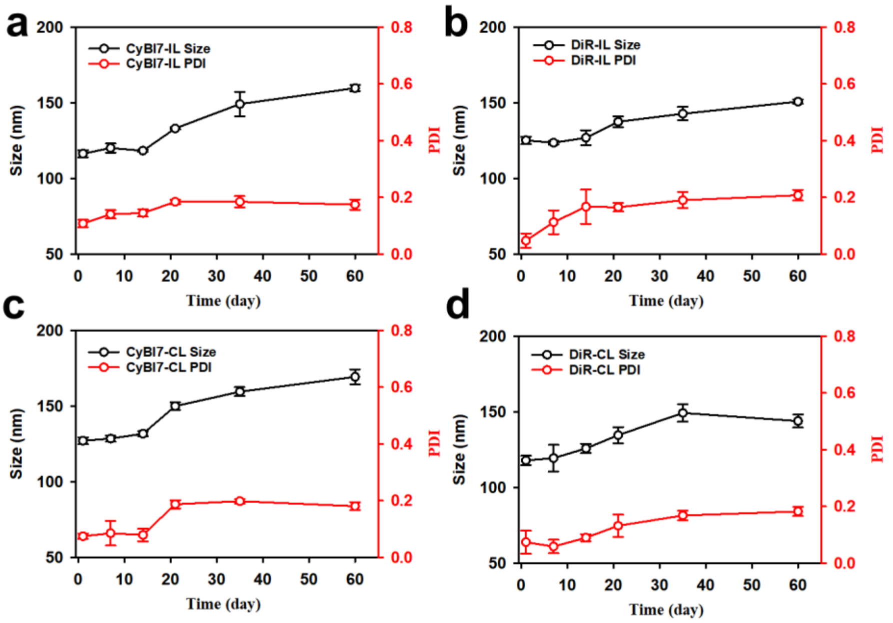

Figure S1. Changes in size and PDI of the liposomes in two months.

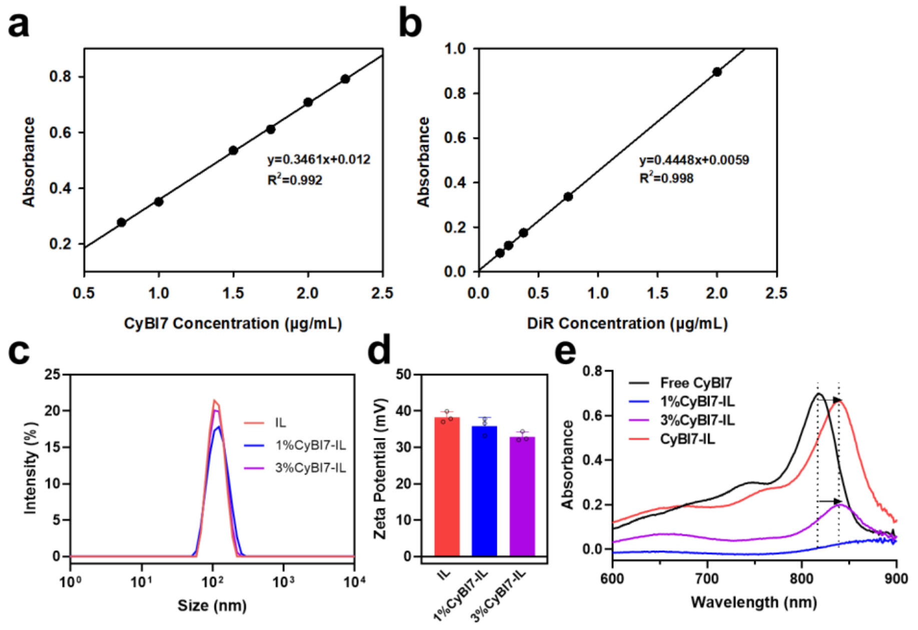

Figure S2. (a-b) Standard curves of absorbance-concentration of CyBI7 and DiR in methanol at 819 nm and 745 nm, respectively. (c-d) Size distributions and zeta potentials of IL, 1% CyBI7-IL and 3% CyBI7-IL in water. (e) NIR absorption spectra of free CyBI7 in methanol, 1% CyBI7-IL, 3% CyBI7-IL and CyBI7-IL in water. Black arrows show the redshift of the maximum absorption.

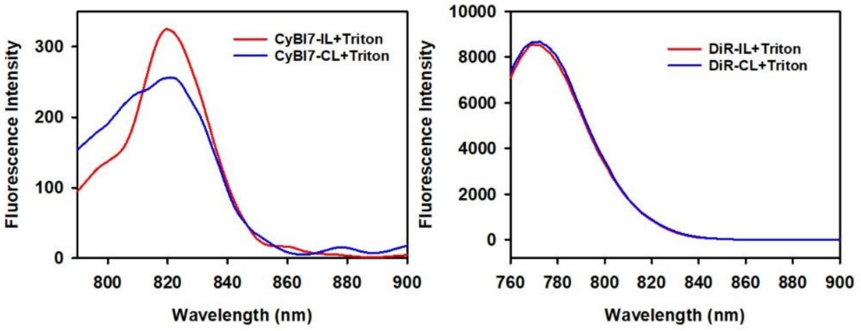

Figure S3. Fluorescence emission spectra of the CyBI7-contained and DiR-contained liposome formulations after disruption by Triton X-100.

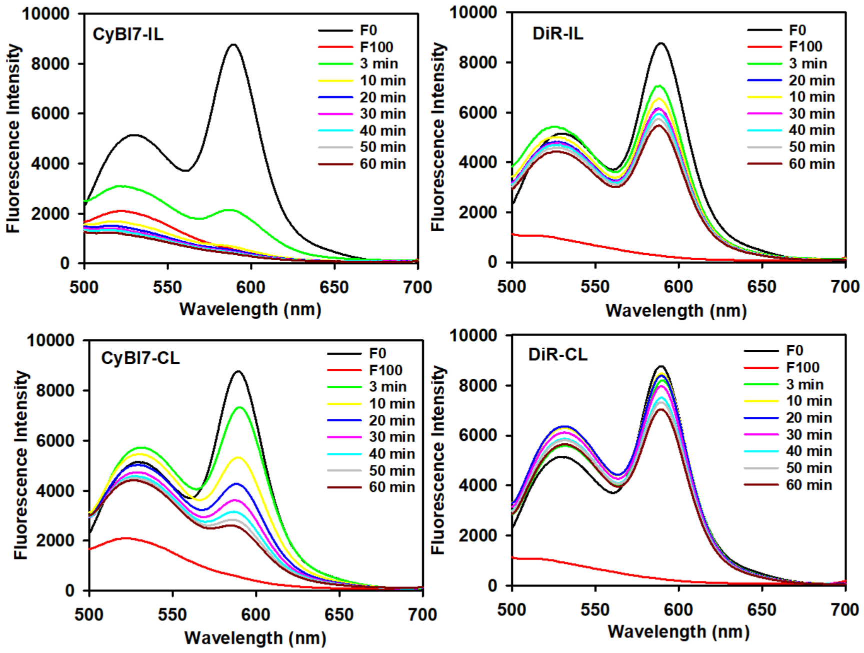

Figure S4. Time-dependent fluorescence emission spectra of FRET-L after mixing with the four liposomes: (a) CyBI7-IL, (b) DiR-IL, (c) CyBI7-CL, (d) DiR-CL.

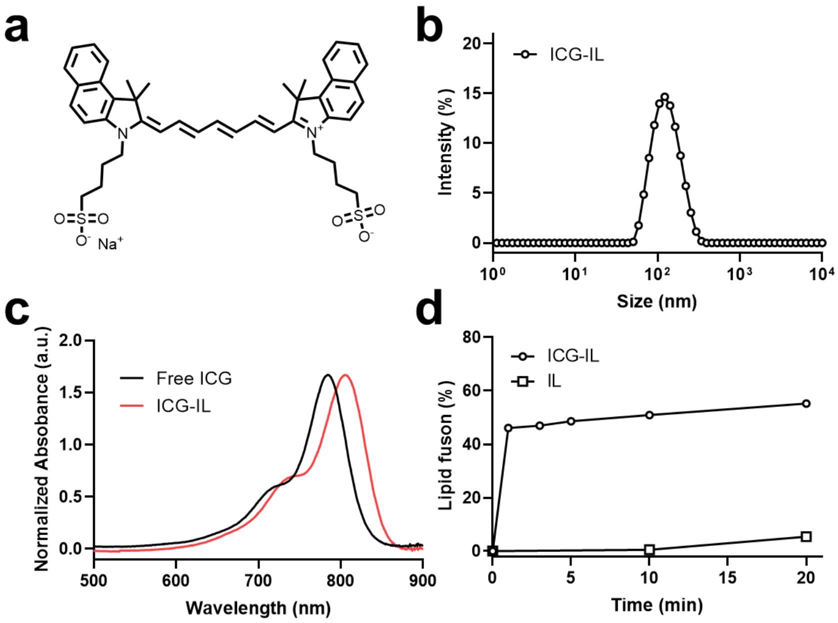

Figure S5. (a) Chemical structure of ICG. (b) Representative size distribution of ICG-IL. (c) Near infrared (NIR) absorption spectra of free ICG and ICG-IL. (d) Normalized lipid fusion kinetics of ICG-IL and IL in NaH_2PO_4 buffer (pH = 7.4).

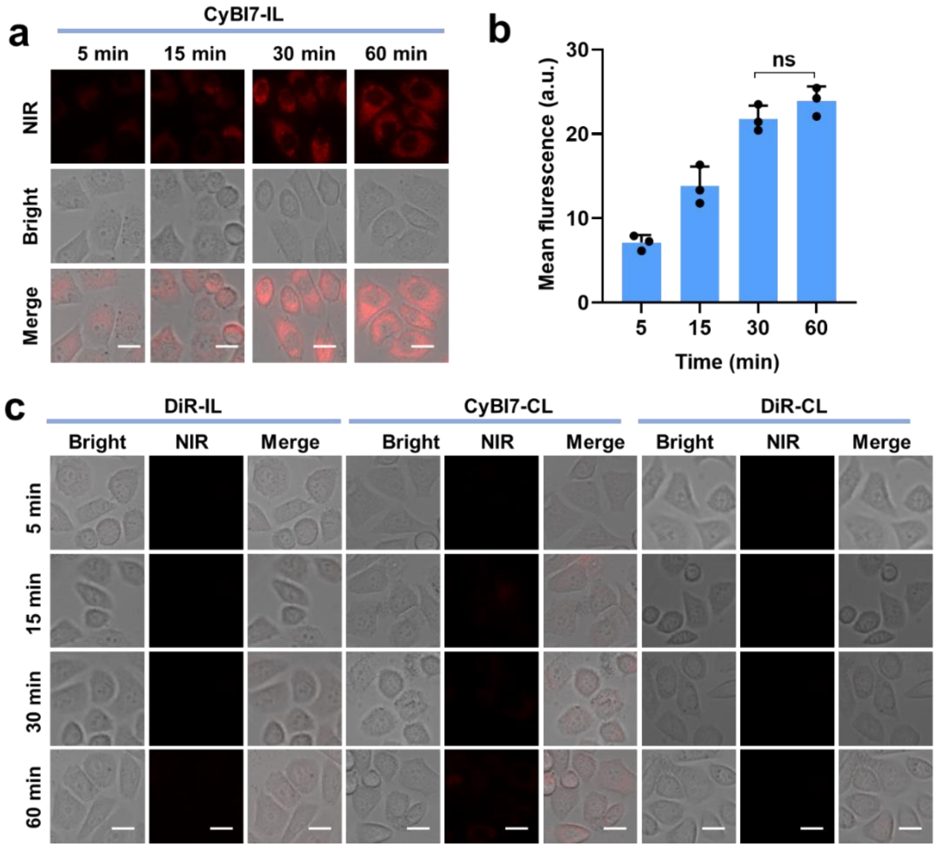

Figure S6. (a) The fluorescence images of cells after incubation with CyBI7-IL for 5 min, 15 min, 30 min and 60 min. Scale bar, 50 μm. (b) The quantitative fluorescence intensity of cells

in the fluorescence images. Statistical significance was calculated via one-way ANOVA with a Tukey test. ns, non-significant. (c) Time dependent cellular uptake of DiR-IL, CyBI7-CL and DiR-CL in BCG-823 cells. Scale bar, 50 µm.

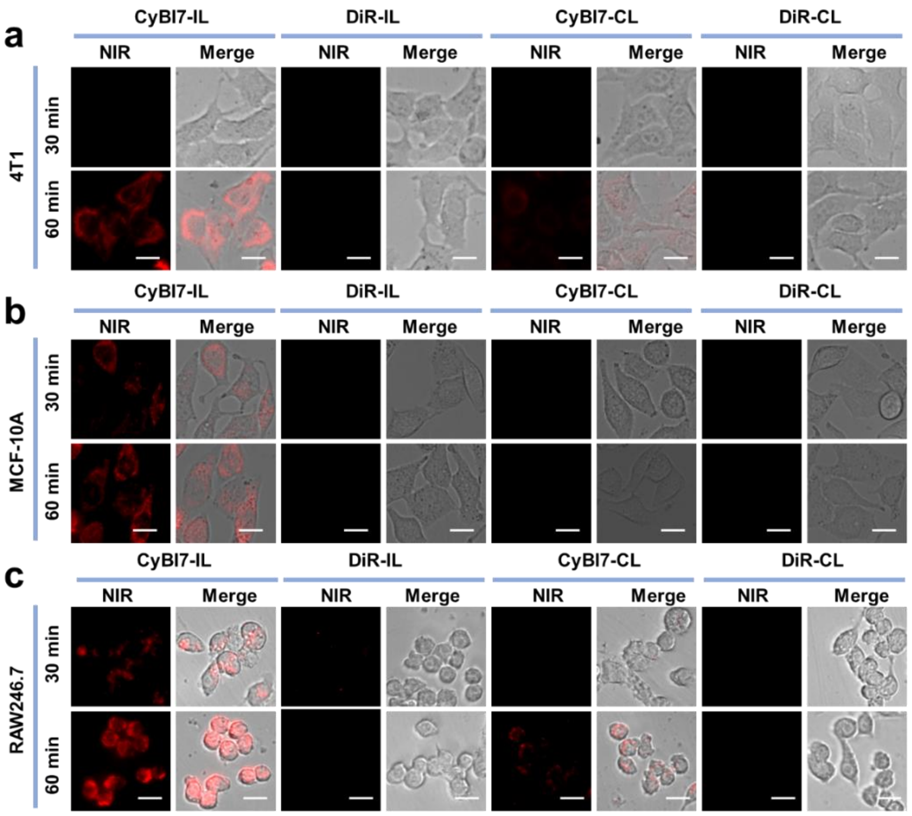

Figure S7. Time dependent cellular uptake of DiR-IL, CyBI7-CL and DiR-CL in 4T1 (a), MCF-10A (b) and RAW246.7 (c) cells. Scale bar, 50 µm.

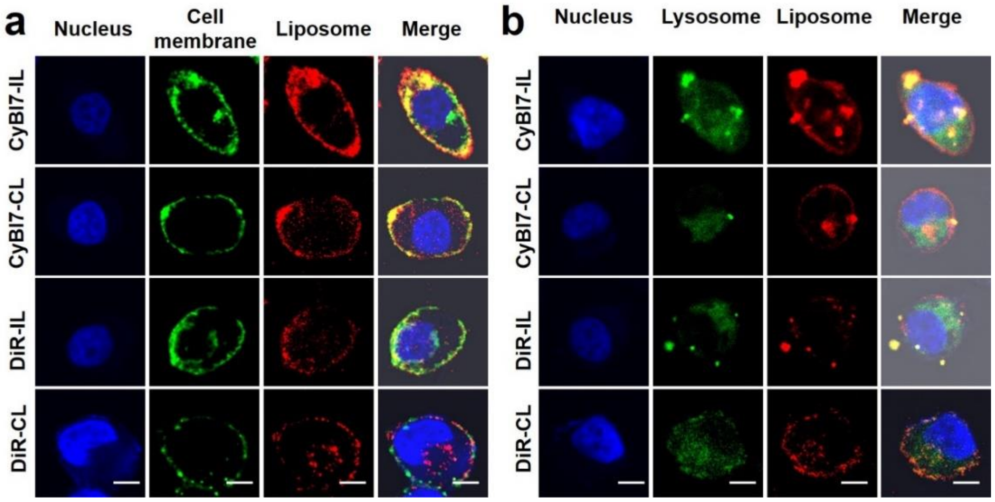

Figure S8. (a) Co-localization of the four liposomes and cell membrane after incubation with BCG-823 cells for 60 min. Scale bar, 20 μm. (b) Co-localization of the four liposomes and lysosomes after incubation with BCG-823 cells for 60 min. Scale bar, 20 μm.

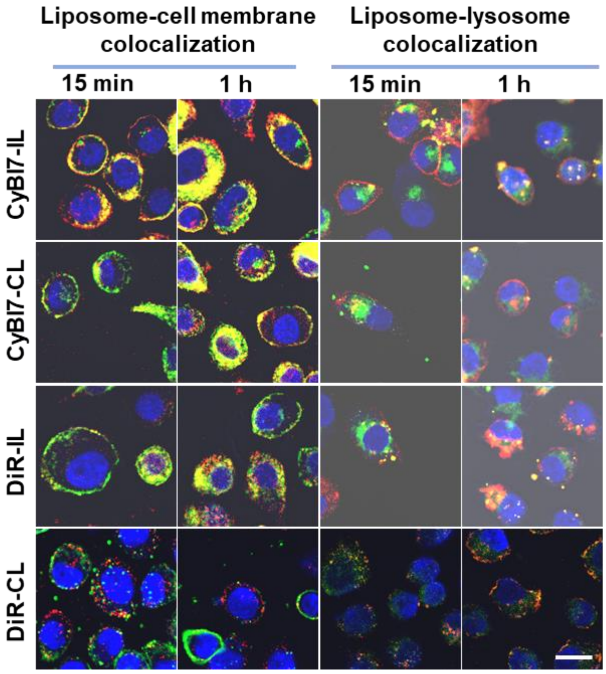

Figure S9. The original liposome/cell membrane or liposome/lysosome colocalization images in Figure 3f, 3g and S8. Scale bar, 20 μm.

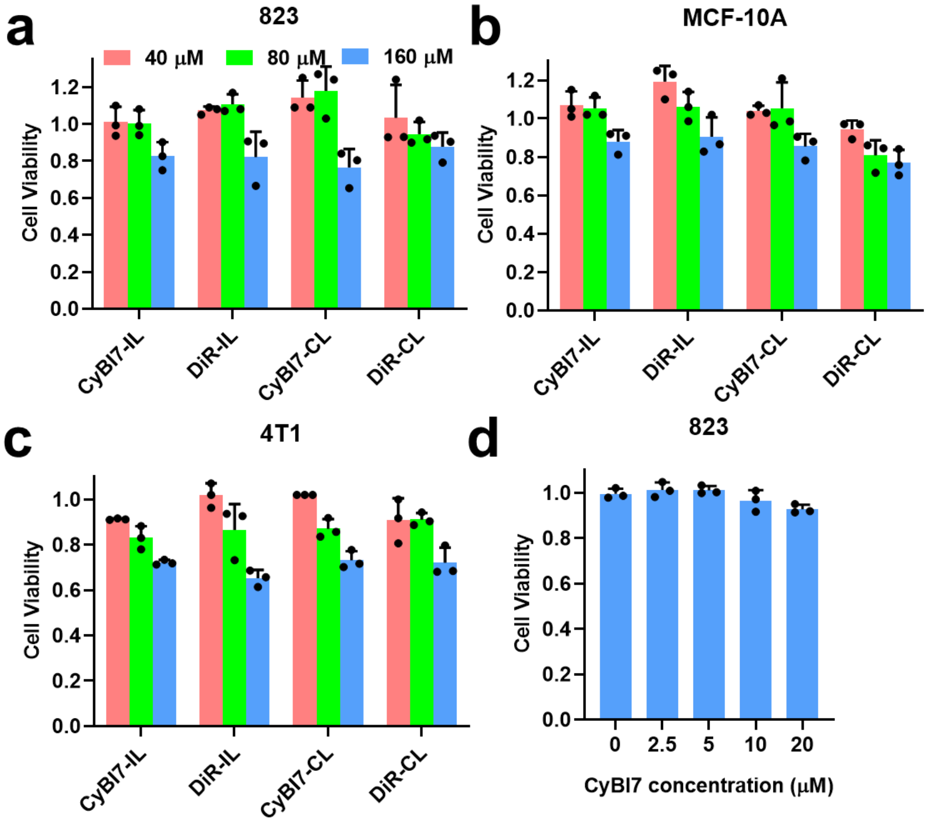

Figure S10. (a-c) Cellular viability of different cells (a. BCG-823 cell, b. MCF-10A cell, c. 4T1 cell) after incubation with the four liposomes for 24 h. (d) Cellular viability of BCG-823 cell after incubation with free CyBI7 for 24 h.

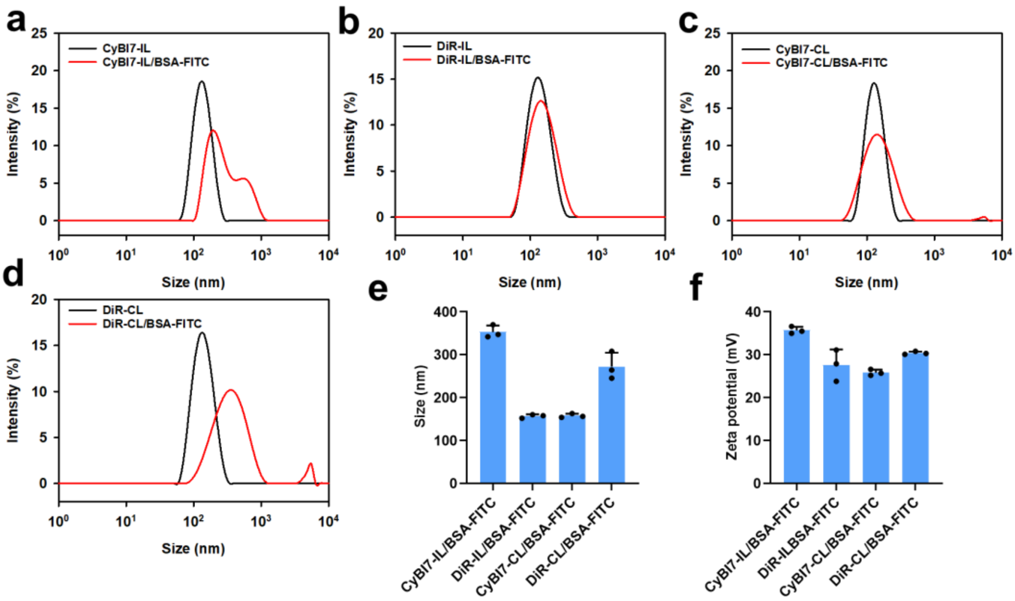

Figure S11. Characterization of liposome/BSA-FITC complexes. (a-d) Size distributions of CyBI7-IL/BSA-FITC, DiR-IL/BSA-FITC, CyBI7-CL/BSA-FITC, DiR-CL/BSA-FITC

complexes. (e) Average size values of the liposome/BSA-FITC complexes. (f) Zeta potentials of the liposome/BSA-FITC complexes.

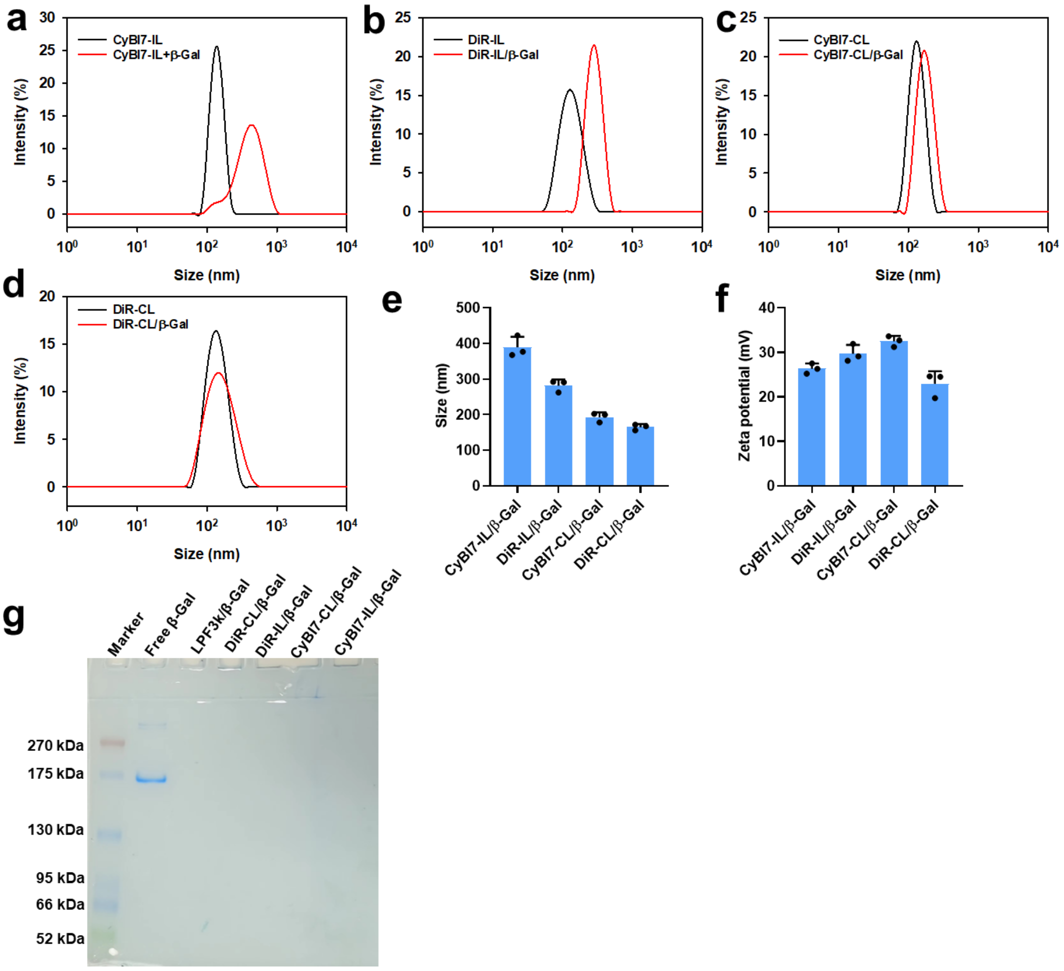

Figure S12. Characterization of liposome/β-gal complexes. (a-d) Size distributions of CyBI7-IL/β-gal, DiR-IL/β-gal, CyBI7-CL/β-gal, DiR-CL/β-gal complexes. (e) Average size values of the liposome/β-gal complexes. (f) Zeta potentials of the liposome/β-gal complexes. (g) The liposome/β-gal complexes and nonassociated proteins were visualized on a native-PAGE gel for the β-gal.

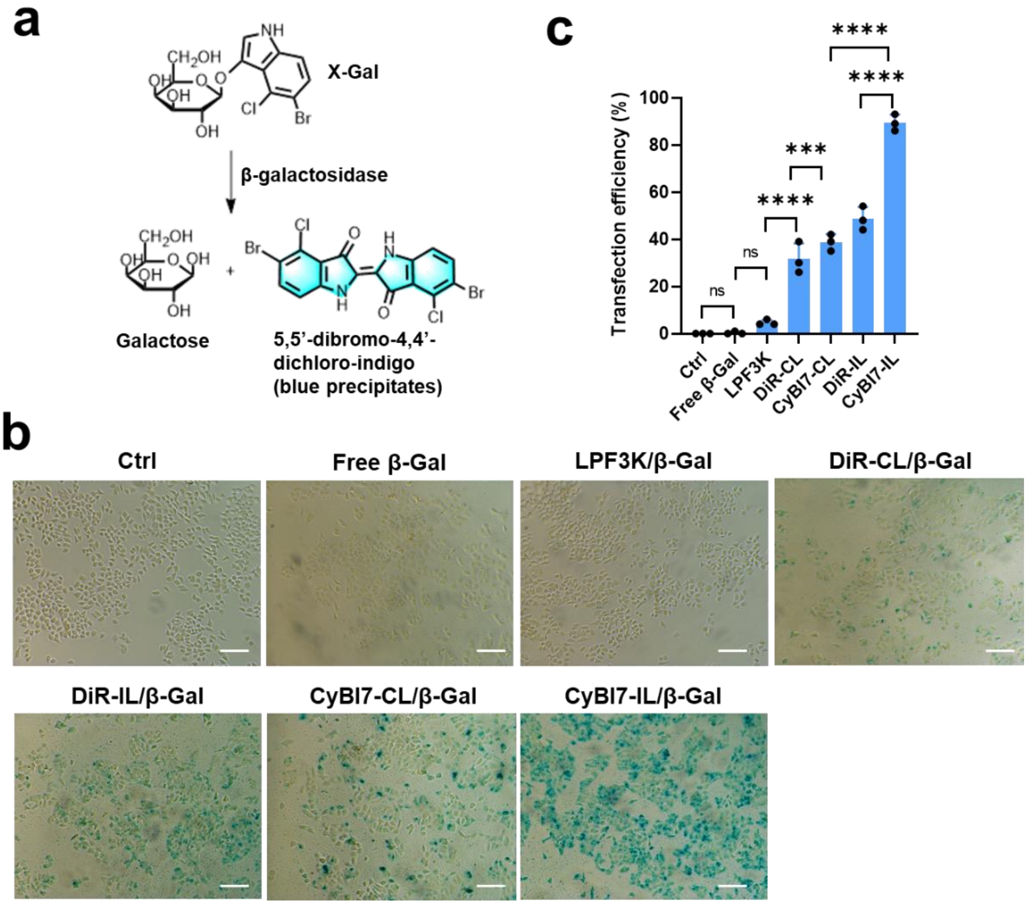

Figure S13. Protein delivery by the liposomes. (a) Schematics showing the chromogenic reaction of X-Gal catalyzed by β-gal. (b) Transfection of β-gal by different carriers in BCG-823 cells. Scale bar, 100 μm. (c) Quantitative transfection efficiency in the β-gal transfection images. Statistical significance was calculated via one-way ANOVA with a Tukey test. *p<0.05, **p<0.01, ***p<0.001, ****p<0.0001.

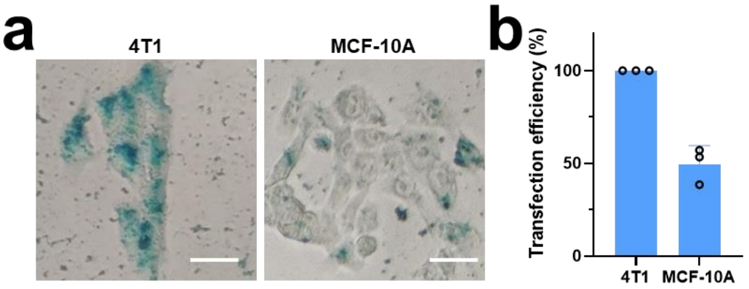

Figure S14. (a) Transfection of β-gal in 4T1 and MCF-10A cells. Scale bar, 20 μm. (b) Quantification of the transfection efficiencies in (a).

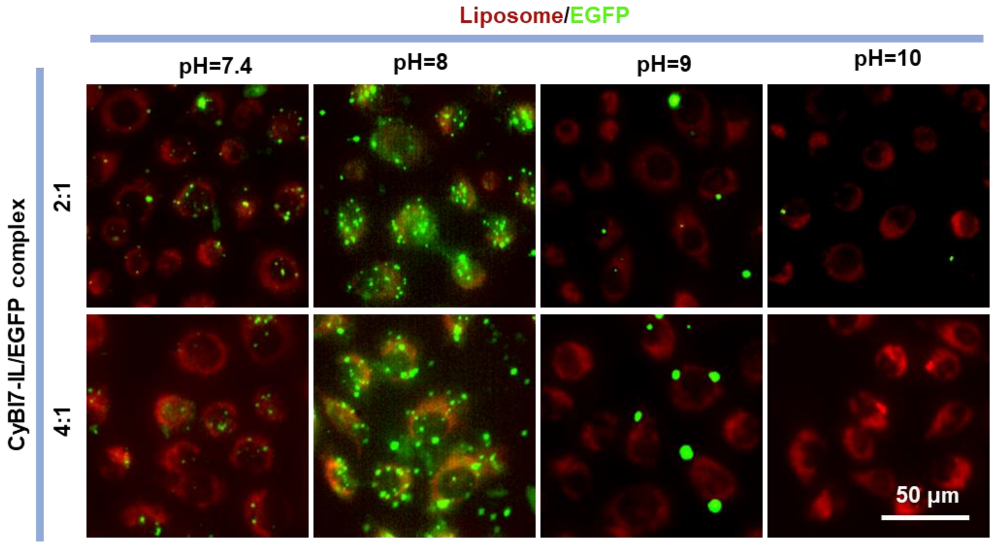

Figure S15. Fluorescence micrographs of BGC-823 cells after transfected with CyBI7-IL/EGFP complexes prepared at different pHs and volume ratios. Scale bar, 50 μm.

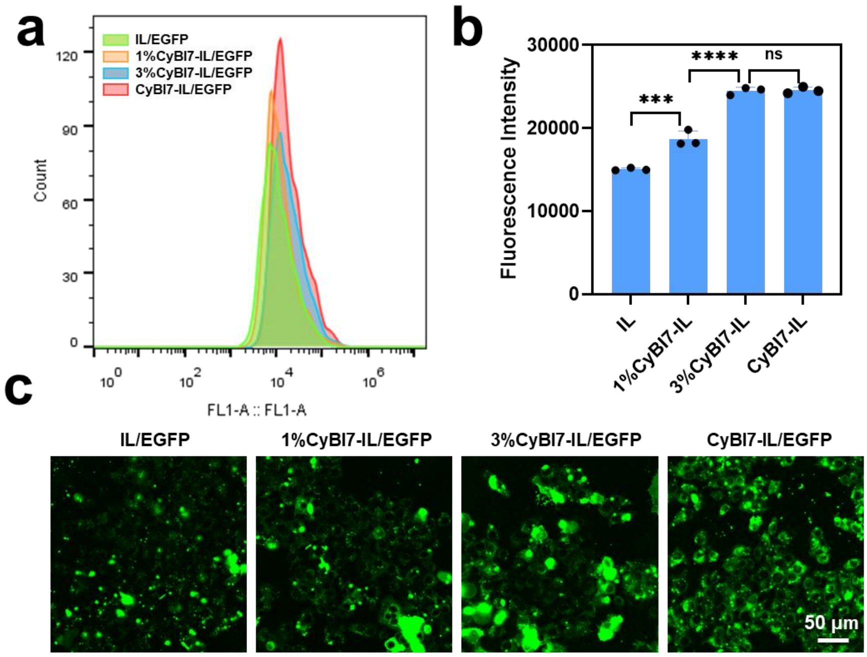

Figure S16. *In vitro* EGFP delivery with CyBI7-IL containing less CyBI7. (a) Flow cytometric histograms of BCG-823 cells incubated with IL/EGFP, 1% CyBI7-IL/EGFP and 3% CyBI7-IL/EGFP complexes for 1 h. 1% CyBI7-IL and 3% CyBI7-IL were liposomes containing less CyBI7, where their dye/lipid feeding ratio were 1% and 3%, respectively. The dye/lipid feeding ratio was 5% for the CyBI7-IL in this study. (b) Quantification of mean fluorescence intensity (MFI) of the treated cells in (a). Data are shown as mean ± SD (n = 3). (c) Fluorescence micrographs of BGC-823 cells treated with IL/EGFP, 1% CyBI7-IL/EGFP and 3% CyBI7-IL/EGFP complexes for 1 h. Scale bar, 50 μm.

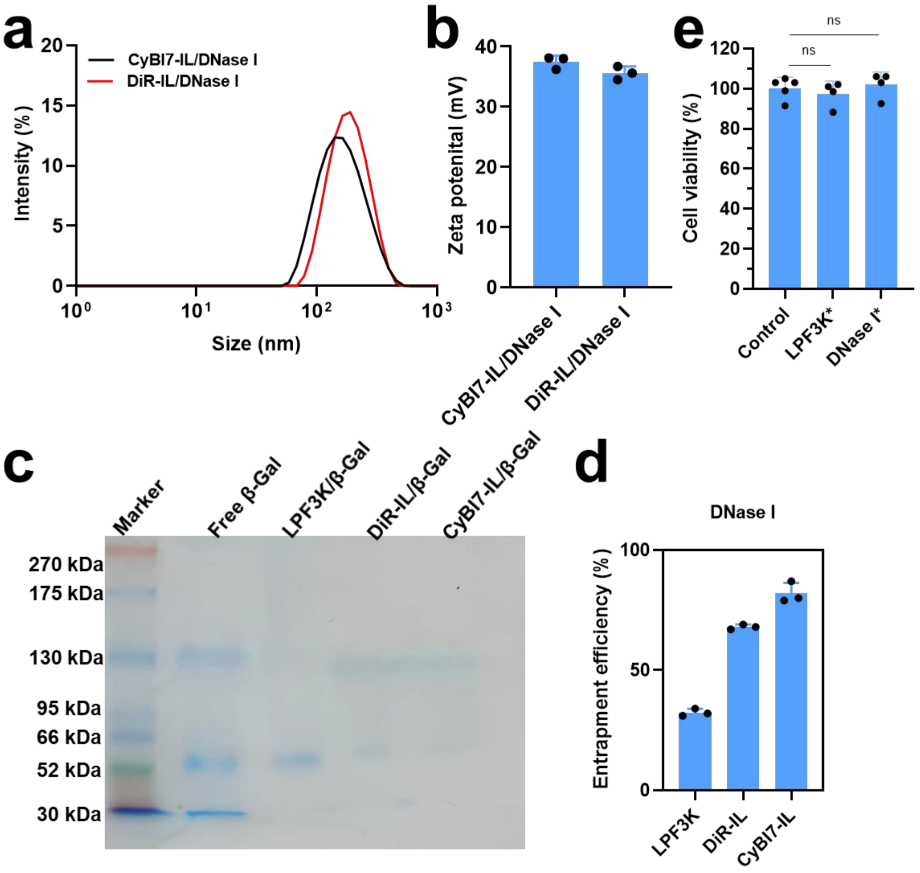

Figure S17. (a) Size distributions of liposome/DNase I complexes. (b) Zeta potentials of liposome/DNase I complexes. (c-d) The liposome/DNase I complexes and nonassociated proteins were visualized on a native-PAGE gel for the DNase I and the quantification (d). (e) The cell viability of BCG-823 cells after incubation with LPF3K alone (1/220 dilution of store solution) and free DNase I alone (24 μg/mL).

Experimental Procedures

Preparation and Characterization of Liposomes with less CyBI7: The liposomes (IL, 1% CyBI7-IL, 3% CyBI7-IL) were prepared with thin-film hydration method. To prepare IL, 1% CyBI7-IL and 3% CyBI7-IL, CyBI7 (0/0.2/0.6 μmol), DOPE (10 μmol) and DOTAP (10 μmol) dispersed in 3 mL of mixed organic solvent (chloroform/methanol, 9/1, v/v) was added into a pear-shaped flask, and the organic solvent was removed by sequential rotary evaporation (15 min) and vacuum desiccation (overnight). Then the dry lipid membrane was hydrated with water (4 mL) to form multilamellar vesicles and the vesicles were further homogenized after extrusion (100 nm polycarbonate, 10 times) under nitrogen. In the end, the homogenized liposomes were dialyzed against water to remove unencapsulated CyBI7 (preparation of IL can

skip this procedure). The sizes and zeta potentials of the liposomes were measured with dynamic light scattering (Malvern ZEN3690). The encapsulation efficiencies of CyBI7 in the liposomes were determined according to the NIR absorbances of CyBI7 (817 nm) and DiR (748 nm) in disrupted liposomes with 0.05% Triton X-100[41]. The loading contents of dyes in the liposomes were the amount of total entrapped dyes divided by the total lipids (mol/mol).

Preparation and Characterization of ICG-IL: To prepare ICG-IL, ICG (1 μmol), DOPE (10 μmol) and DOTAP (10 μmol) dispersed in 3 mL of mixed organic solvent (chloroform/methanol, 9/1, v/v) was added into a pear-shaped flask, and the organic solvent was removed by sequential rotary evaporation (15 min) and vacuum desiccation (overnight). Then the dry lipid membrane was hydrated with water (4 mL) to form multilamellar vesicles and the vesicles were further homogenized after extrusion (100 nm polycarbonate, 10 times) under nitrogen. In the end, the homogenized liposomes were dialyzed against water to remove unencapsulated ICG. The sizes and zeta potentials of the liposomes were measured with dynamic light scattering (Malvern ZEN3690).

Fusogenic efficiency of IL and ICG-IL: Then, the liposomes (IL, ICG-IL, 90 μL) were mixed with FRET-L (2 mM, 10 μL) and diluted in phosphate buffer (10 mM, pH = 7.4, 500 μL) and the recovery of fluorescence energy transfer (FRET) between NBD-PE and Rho-PE (Ex 475 nm, Em 500-700 nm) was tracked at different times (0-20 min) and after addition of Triton X-100 (0.1%) to monitor the membrane fusion process. The liposome mixture containing Trition X-100 mimics the FRET effect after 100% fusion. The fusion efficiency was calculated according to the following formula:

$$\eta = (R_t - R_0)/(R_{100} - R_0) \times 100\%$$

where R_t was the acceptor/donor fluorescence ratio (Ex 475 nm, Em 592 nm/Em 516 nm) of FRET-L/liposome mixture at different times, R_0 was the ratio of FRET-L (2 mM, 10 μL) in phosphate buffer (590 μL) and R_{100} was the fluorescence intensity of liposome mixture containing Triton X-100.

Cellular Uptake of Liposomes in other cell lines: 4T1 cells were cultured in RPMI-1640 media (10% FBS, 1% P/S) at 37 °C in 5% CO_2 atmosphere. MCF-10A cells were cultured in DMEM media (10% FBS, 1% P/S) at 37 °C in 5% CO_2 atmosphere. The cells (4T1 and MCF-10A) were treated with serum-free media containing CyBI7-IL (40 μg mL^{-1} lipids, 100 μL/well) for different times (5, 10, 20, 30, 60 min). Then the cells were washed with PBS and treated with fresh cell media before fluorescence imaging (Leica).

Polyacrylamide gel electrophoresis of liposome/β-gal complexes. The liposome/β-gal complexes were prepared with β-gal (5 mg/mL, 2.2 μL) and lipsomes (4 mM, 20 μL) in water.

After 15 minutes incubation at room temperature, the preparation were further mixed with 4.4 μl protein loading buffer (Promega, Madison, WI). Migration was performed on a nondenaturing polyacrylamide gel (6%) for 2 hours at 160 V and the β-gal protein revealed by Coomassie blue staining.

β-Gal Staining in Situ in other cells: The 4T1 cells and MCF-10A cells were seeded in 96-well plates (5000 cells/well) and when the cell confluency reached 80%, the cells were treated with CyBI7-IL/β-gal complexes (6 μg mL^{-1} β-Gal) for 60 min. Then the cells were washed with PBS and stained according to the manufacturer's protocol. Finally, the intracellular distribution of β-Gal was observed under bright field.

Cytotoxicity of the liposome formulations: The cells (BCG-823, 4T1, MCF-10A) were seeded on 96-well plates with a density of 4000 cells/well. When the cell confluency reached 60-70%, the cell medium was removed and the cells were incubated with serum-free medium containing liposomes (CyBI7-IL, DiR-IL, CyBI7-CL, DiR-CL) with different concentrations (0, 40, 80, 160 μM) for 4 h (37 °C, 5% CO_2). Then, the medium was replaced with cell culture medium and further incubated for 20 h (37 °C, 5% CO_2). In the end, the cells were treated with 10% CCK-8 for 3 h (37 °C, 5% CO_2) and the cell viabilities were evaluated according to the absorbances at 450 nm.

Transfection of EGFP at different pHs: The liposome/EGFP complexes were prepared at different volume ratios (4/1, 2/1, 1/1) and different pHs (pH = 7.4, 8, 9 or 10). In pH = 8 and volume ratio = 4/1, the CyBI7-IL (2 mM, 4 μL) was mixed with EGFP (1 mg/mL, 1 μL) and phosphate buffer (10 mM, pH =8, 4 μL). The mixture was incubated for 15 min and further diluted with serum-free media and added into seeded BGC-823 cells in 96-well plates. After incubation with the mixture for 1 h, the cells were observed under fluorescence microscope to check the transfection of EGFP. The transfection of EGFP in other conditions was performed similarly.

Polyacrylamide gel electrophoresis of liposome/DNase I complexes. The liposome/DNase I complexes were prepared with DNase I (5 mg/mL, 2.2 μL) and liposomes CyBI7-IL (6 mM, 20.5 μL), DiR-IL (6 mM, 20.5 μL) or LPF3k (P3000, 11 μL, LPF 3k 8.25 μL) in water. After 15 minutes incubation at room temperature, the preparation were further mixed with 4.4 μl protein loading buffer (Promega, Madison, WI). Migration was performed on a nondenaturing polyacrylamide gel (6%) for 50 min at 160 V and the DNase I was revealed by Coomassie blue staining. The quantification of encapsulated efficiency was determined by comparing the band intensity of the dimers (~ 58 kDa).
